# A Calculation Method for Surface Energies with Thermodynamic Characteristics and Its Application in Investigating Activity Mechanisms for Nanoporous W

**DOI:** 10.3390/ma18214895

**Published:** 2025-10-26

**Authors:** Yingtong Guo, Kai Wang, Xingyu Chen, Xin Chen, Zumin Wang, Yuan Huang

**Affiliations:** Institute of Advanced Metallic Materials, School of Materials Science and Engineering, Tianjin University, Tianjin 300350, China

**Keywords:** first-principles calculations, surface energy, nanoporous W, origin of activity

## Abstract

Surface energy is involved in various thermodynamic processes, providing a driving force for thermodynamic reactions. However, surface energies applied in current engineering calculations are generally measured in J/m^2^, which is unsuitable for thermodynamic analysis. To solve this problem, the calculation formula for surface energies was modified to convert the unit of measurement, transforming the non-thermodynamic measurement unit J/m^2^ into the thermodynamically characterized kJ/mol. The calculated surface energy values measured in kJ/mol are unstable due to the influence of the number of atomic layers (*t*) in the constructed models. Meanwhile, the problem of determining the surface layer thickness, i.e., the number of atomic layers with surface characteristics (*t*_0_), remains unresolved in surface science. Therefore, the extended Finnis Sinclair (EFS) potential was improved by extending the nearest neighbor range and utilized in analyzing the energy per atom, resulting in the determined number of *t*_0_. These results suggest that selecting the surface layer number corresponding to the first to third nearest-neighbor atoms could be appropriate, and the resulting surface energies in kJ/mol appear reasonable. The validity of this computational method and the origin of nanoporous W activity were confirmed by analyzing the changes in total surface energy before and after nano-treatment using the novel nanosized approach.

## 1. Introduction

Nano-Porous Metals (NPMs) and nano-porous oxides exhibit unique physicochemical characteristics that make them useful in energy storage and catalysis [1,2]. For example, the hybrid structure of nanoporous gold and nanocrystalline MnO_2_ can serve as active electrode materials for electrochemical supercapacitors [1]. Additionally, nanoporous tungsten oxide film can be used as a visible light-driven photocatalyst [3]. Among NPMs, Nano-Porous W (NPW) is a promising material for a wide range of applications with a large specific surface area and high surface activity [4,5], such as supporting Te metal to serve as a cathode material with high specific volumetric capacity and excellent cycling performance [6]. In addition, W/Cu layered composites prepared with W as the matrix material and Cu as the surface material can be utilized as Plasma Facing Materials (PFMs) and electronic packaging materials. However, the W-Cu binary system is a binary immiscible system with positive formation enthalpy, and the significant differences in physical properties between W and Cu lead to challenges in constructing a metallurgical bonding interface and fabricating W/Cu layered composites. To address this, nano-treatment was carried out to obtain nanoporous structures on the W surface, which would enhance surface activity. This enhancement in surface activity provides the thermodynamic driving force necessary for direct alloying between W and Cu, facilitating the successful preparation of W/Cu layered composites with a metallurgical bonding interface [7]. Thus, the high surface activity of NPW is crucial for promoting direct alloying between W and Cu.

It was found that the improvement of surface energy is a predominant factor in the enhancement of surface activity of NPW due to the exposure of crystal planes with high surface energies, which generally occurs after nano-treatment [8,9]. Therefore, the activity mechanism of NPMs can be investigated by analyzing the total surface energy and that of different crystal planes before and after nano-treatment [7,10]. For the binary immiscible W-Cu system with positive formation enthalpy, surface energy can serve as the primary thermodynamic driving force, providing the initial energy for W-Cu direct alloying. According to thermodynamic principles, publications, and literature [11,12], the basic unit of measurement for thermodynamic driving forces and material thermodynamic calculations should be J/mol (or kJ/mol, cal/mol). However, the conventional unit for surface energy is J/m^2^. Moreover, no material thermodynamic calculations with J/m^2^ as the unit have been observed. To overcome the limitation that surface energies in units of J/m^2^ cannot be involved in thermodynamic calculations and analysis, the unit of measurement for surface energies should be converted, transforming the non-thermodynamically characterized J/m^2^ into the thermodynamically characterized kJ/mol, so that surface energies can be incorporated into thermodynamic calculations.

Currently, surface energy measured in J/m^2^ is calculated using first-principles methods [13]. The calculation process involved the construction of a surface slab model and the calculation of the energy difference between the surface slab model and the bulk model. Finally, the energy difference is divided by the surface area of both sides of the slab model. This work proposes that the conversion of unit of measurement from J/m^2^ to kJ/mol requires modification of the denominator in the surface energy calculation formula, transforming the surface area on both sides into the molar number of atoms in the surface slab model. Meanwhile, the non-first-principles methods for calculating the average molar surface energy of materials are based on the energy of the atoms in the “surface layer” divided by the mole number of atoms, similarly [14]. It is clear that the dependence of surface energy values on the mole number of atoms in the surface commonly exists. As a result, the range of the surface layer and the corresponding mole number should be determined. To solve these problems, the surface energies of various crystal planes are investigated in different units of measurement, with a focus on identifying the influencing factors and computational methods for surface energies in kJ/mol. By decomposing the first-principles calculation formula in detail, it is confirmed that the number of atomic layers, *t*, is the factor influencing the calculated surface energy value in kJ/mol. To give a selection scheme for the number of atomic layers, the Extended Finnis Sinclair (EFS) potential for W metal [15,16] can be improved and applied in calculations for the energy per atom, thus the optimal nearest-neighbor range and the corresponding number of atomic layers in the surface layer were selected. Depending on this selection scheme, suitable surface energy values of the (*hkl*) crystal planes measured in kJ/mol ($E_{\mathrm{surf}(M)}^{hkl}$), as well as the average surface energy of materials ($E_{\mathrm{surf}(M)}$), can be obtained. At the same time, the universality of the surface layer selection method was confirmed through fitting calculations of potential functions for other metals, thereby validating the calculation criterion for surface energies with thermodynamic characteristics. This calculation criterion for molar surface energies can serve as a reference for other metal systems besides W. Eventually, the average surface energies of W metal measured in kJ/mol before and after treatment using the novel nanosized method can be obtained by combining the XRD patterns of the metal surface with surface energies calculated using the aforementioned method. Therefore, this work could provide a theoretical reference for studies on the mechanism of the activity of NPMs.

## 2. Materials and Methods

### 2.1. Surface Nano-Treatment of W Metal

In this work, a nanoporous structure was synthesized on the W surface by a novel nanosized method based on anodization of a F^−^-containing electrolyte and deoxidation annealing in a H_2_ atmosphere. For example, to fabricate the nanoporous layer on the W surface, the W foil was first ground and polished and then subjected to anodic oxidation, which was carried out in an electrolyte containing 1 mol/L Na_2_SO_4_ and 0.5 wt% NaF [17] under a constant temperature water bath. Using the DH1716A-6 DC stabilized power supply (DH1716A-6, Beijing Dahua Electronic Instrument Co., Ltd., Beijing, China), anodization was performed at 50 V and 20 °C for 7 min, resulting in a nanoporous layer consisting of tungsten oxide (blue-violet colored, denoted as WO*_x_*, *x* = 2.72–2.90) [18] on the W surface. However, when the anodized W is utilized in the production of W/Cu layered composites, the presence of oxygen inside the nanoporous structure is unfavorable for W-Cu alloying. Consequently, the prepared anodized W foils were placed in a tube-type annealing furnace (OTL-1200, Nanjing Nanda Instrument Co., Ltd., Nanjing, China) and subjected to heating in a H_2_ atmosphere for a nanoporous pure tungsten layer that was almost oxygen-free. Comparison results show that constant temperature annealing at 600 °C for 3 h can lead to the optimal structure on the W surface.

### 2.2. Characterization of the Structure and Activity of the Nanoporous Layer on the W-Metal Surface

The surface and cross-sectional morphology of the W nanoporous structures were examined by a thermal field emission scanning electron microscope (JSM-7800F, JEOL Ltd., Tokyo, Japan). Meanwhile, the equipped energy dispersive spectrometer (EDS) detector was used to characterize the chemical composition. In addition, XRD analysis was utilized to analyze the crystal structure of the W surface before and after nano-treatment. Tests were conducted on a Bruker Advance D8 diffractometer (D8 Advance, Bruker AXS, Karlsruhe, Germany) with Cu*K*_α_ radiation (*λ* = 1.5406 Å), scanning in the 2*θ* range from 20° to 90° at the rate of 4°/min.

The electrochemical tests were conducted on an electrochemical workstation (CHI660E, CH Instruments, Austin, TX, USA) to compare electrochemical activity before and after nano-treatment. A conventional three-electrode system was used, in which the W sample with a working area of 2 cm^2^, the platinum plate, and the saturated calomel electrode (SCE) served as the working electrode, counter electrode, and reference electrode, respectively. All the electrodes were immersed in a 0.5 M H_2_SO_4_ solution. The detailed parameters are depicted below: (1) The linear sweep voltammetry (LSV) experiments were carried out from −1.0 V to 0 V vs. SCE at a scanning rate of 5 mV/s; (2) Electrochemical impedance spectroscopy (EIS) measurements were applied at the potential of −0.56 V vs. SCE, with the test frequencies ranging from 10^5^ to 0.1 Hz; (3) The electrochemical active surface area (ECSA) can be estimated by the double-layer capacitance $C_{\mathrm{dl}}$. In this work, $C_{\mathrm{dl}}$ values were obtained by cyclic voltammetry (CV) tests in the non-faradic potential region (±50 mV vs. OCP).

It should be mentioned that the potentials vs. SCE ($E_{\mathrm{vs}.\mathrm{SCE}}$) were normalized to the reversible hydrogen electrode (RHE) ($E_{\mathrm{vs}.\mathrm{RHE}}$) via the Nernst equation (Equation (1)) [19]. Moreover, this work compared the as-obtained potentials without IR compensation, where *I* is the current and *R* is the total resistance.

(1) $$E_{\mathrm{vs}.\mathrm{RHE}}=E_{\mathrm{vs}.\mathrm{SCE}}+0.260\;V$$

### 2.3. Calculation and Analysis for Surface Energies in Different Units of Measurement

Since the surface energy of a specific W crystal plane is challenging to determine by experiments according to previous research [20,21], the surface energies of W metal before and after nano-treatment were analyzed by combining calculations of surface energies for different crystal planes with XRD tests on the metal surface. First, XRD tests were performed to obtain the XRD patterns, from which the ratio of crystal planes ($f_{hkl}$) before and after nano-treatment can be determined. Then, first-principles calculations were adopted to calculate the surface energy of each W crystal plane ($E_{\mathrm{surf}}^{hkl}$) in different units of measurement using the Cambridge Serial Total Energy Package (CASTEP) module in the Materials Studio 2020 software, including $E_{\mathrm{surf}(s)}^{hkl}$ (in J/m^2^) and $E_{\mathrm{surf}(M)}^{hkl}$ (in kJ/mol) obtained from the modified surface energy calculation formula. Eventually, $E_{\mathrm{surf}(M)}$, the average molar surface energy of W before and after nano-treatment, was calculated and compared according to the equation $E_{\mathrm{surf}}=\sum_{hkl}f_{hkl}E_{\mathrm{surf}}^{hkl}$ [7], where $E_{\mathrm{surf}}^{hkl}$ was selected to imply $E_{\mathrm{surf}(M)}^{hkl}$.

**Figure 1:**
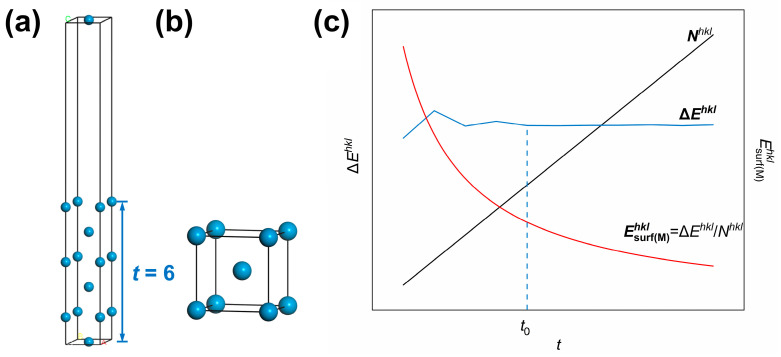
First-principles method for surface energy calculations: (**a**) surface slab model and (**b**) bulk model of W metal. *t*—number of atomic layers for the surface slab model); (**c**) dependence of the mole number ($N^{hkl}$), the energy difference ($\Delta E^{hkl}$), and the calculated value of surface energy in kJ/mol ($E_{\mathrm{surf}(M)}^{hkl}$) according Equation (5) on *t* in the surface slab model of the (*hkl*) crystal plane. The different colored letters in (**a**) represent labeled axes, defining the crystal directions corresponding to the atomic arrangements in the model.

The first-principles computational method of the surface energy $E_{\mathrm{surf}}^{hkl}$ is described as follows. Surfaces could be created by cleavage along specific crystal planes in the bulk material. Thus, the amount of energy difference before and after cleavage is equivalent to the energy required to create two surfaces [13]. The surface energy expressed in J/m^2^, denoted as $E_{\mathrm{surf}(s)}^{hkl}$, can be calculated as follows. Subtracting the energy of the bulk model with the same atomic number as the slab model from the total energy of the surface slab model, the difference is referred to as the numerator. At the same time, the denominator is twice the cross-sectional area of the surface slab model. The W conventional unit cell, also called the bulk model, is cleaved along the (*hkl*) crystal plane, followed by adding a vacuum layer in the *z*-axis direction to prevent periodic interactions. The resulting model is the surface slab model of the (*hkl*) crystal plane. The schematic diagrams of the surface slab model and bulk model are displayed in Figure 1a and Figure 1b, respectively. Subsequently, the total energies were calculated for the surface slab model and the W conventional unit cell for all atoms, unrelaxed and fully relaxed to the equilibrium state. Calculations were performed using ultrasoft pseudopotentials and the Perdew-Burke-Ernzerhof (PBE) functional based on the generalized gradient approximation (GGA). In the meantime, the Broyden-Fletcher-Goldfarb-Shanno (BFGS) method was used for geometry optimization, with the SCF convergence tolerance set to 1 × 10^−6^ eV/atom.

For the (*hkl*) crystal plane, the surface energy value $E_{\mathrm{surf}(s)}^{hkl}$ can be determined using Equations (2) and (3), in units of J/m^2^.

(2) $$E_{\mathrm{surf}(s)}^{hkl}\left[J\cdot m^{-2}\right]=\frac{1}{2}\times \frac{E_{\mathrm{slab}}^{hkl}-n^{hkl}\cdot E_{\mathrm{bulk}}}{S}$$

(3) $$n^{hkl}=\frac{n_{\mathrm{slab}}^{hkl}}{n_{\mathrm{bulk}}}=\frac{n_{\mathrm{slab}}^{hkl}}{2}$$

In Equations (2) and (3), $E_{\mathrm{slab}}^{hkl}$ is the total energy of the (*hkl*) surface slab model, while $E_{\mathrm{bulk}}$ is the energy of the bulk W (bulk model, i.e., the conventional unit cell adopted in this method). The surface area, denoted as $S^{hkl}$, is the contact surface area between the vacuum and outermost atomic layers. $n_{\mathrm{slab}}^{hkl}$ is the total number of atoms in the (*hkl*) surface slab model. The quantity of atoms in the bulk W is represented by the variable $n_{\mathrm{bulk}}$, which equals 2 for the W conventional unit cell. Therefore, $n^{hkl}$ represents the multiplicative relationship between the number of atoms in the surface slab and bulk models.

The unit J/m^2^ (corresponding to energy per unit area) is a unit of measurement for non-thermodynamic characteristics. To overcome the limitation of surface energies in J/m^2^ in thermodynamic analysis, the unit of measurement was converted to thermodynamic-characterized kJ/mol (corresponding to energy per mole). At this point, Equation (2) can be modified into Equation (4), then the surface energies in units of J/m^2^ ($E_{\mathrm{surf}(s)}^{hkl}$) are converted into those in units of kJ/mol ($E_{\mathrm{surf}(M)}^{hkl}$).

(4) $$E_{\mathrm{surf}(M)}^{hkl}[\mathrm{kJ}\cdot {\mathrm{mol}}^{-1}]=\frac{E_{\mathrm{slab}}^{hkl}-n^{hkl}\cdot E_{\mathrm{bulk}}}{n_{\mathrm{slab}}^{hkl}/N_{A}}\cdot C$$

where in $N_{A}$ is the Avogadro constant and C (=1.60 × 10^−19^ J/eV) is the conversion factor from electron volt (eV) to joule (J). Making $\Delta E^{hkl}=E_{\mathrm{slab}}^{hkl}-n^{hkl}E_{\mathrm{bulk}}$ and $N^{hkl}=n_{\mathrm{slab}}^{hkl}/N_{A}$, Equation (4) can be transformed into Equation (5):

(5) $$E_{\mathrm{surf}(M)}^{hkl}=\Delta E^{hkl}/N^{hkl}$$

It can be seen from Equation (5) that surface energies in units of kJ/mol is actually obtained by dividing the formation energy for surfaces by cleavage $\Delta E^{hkl}$ by the mole number of atoms in the surface slab model $N^{hkl}$. Since $N^{hkl}$ is the product of $N_{0}^{hkl}$, the number of atoms within a monolayer and *t*, the number of atomic layers, the dependence of $E_{\mathrm{surf}(M)}^{hkl}$, $\Delta E^{hkl}$ and $N^{hkl}$ on *t* can be analyzed as below. Taking the W(110) crystal plane as an example, the curves of $E_{\mathrm{surf}(M)}^{hkl}$, $\Delta E^{hkl}$ and $N^{hkl}$ as a function of the number of atomic layers in the surface slab model (*t*) are plotted in Figure 1c. It can be seen in the plot that $\Delta E^{hkl}$ fluctuates and then stabilizes as *t* increases and finally converges to a stable value after $t>t_{0}$. Meanwhile, $N^{hkl}$ has been monotonically increasing with *t*. As a result, the surface energy of the (*hkl*) crystal plane in units of kJ/mol ($E_{\mathrm{surf}(M)}^{hkl}$) experiences a reduction with the rise of *t*, so a stable value of $E_{\mathrm{surf}(M)}^{hkl}$ cannot be obtained.

(6) $$E_{\mathrm{surf}(M)}[\mathrm{kJ}\cdot {\mathrm{mol}}^{-1}]=\frac{S\cdot \gamma^{S,0}}{\rho \cdot \Delta V/M}$$

As mentioned in the introduction, the average molar surface energy within a specific surface range of the material, regarded as $E_{\mathrm{surf}(M)}$, measured in kJ/mol, can be calculated by a non-first-principles method in addition to the first-principles method described above. Equation (2) is the commonly used formula for the average surface energy of materials in kJ/mol [14].

**Figure 2:**
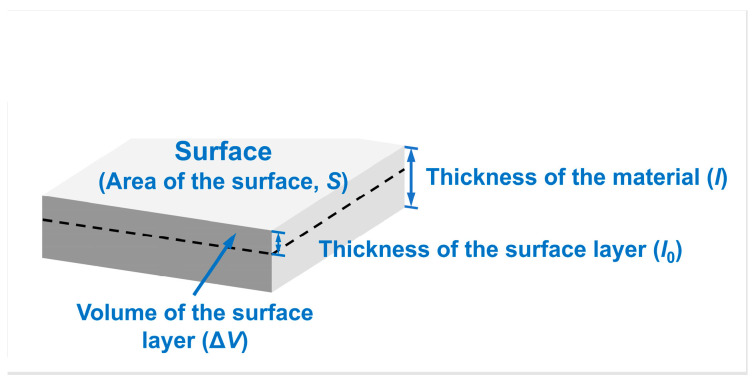
Non-first-principles computational model for average molar surface energies measured in kJ/mol. The black dashed line delineates between the surface and interior of the material. The layer of the material above the black dashed outline represents the “surface layer”. S—area of the surface, l—thickness of the material, $l_{0}$—thickness of the surface layer, ΔV—volume of the surface layer.

Taking W metal as an example, the variable S is the surface area of the W sample, $\gamma^{S,0}$ is the specific surface energy of W at 0 K, and ΔV is the volume of the surface layer providing surface energy for the system. Moreover, M and ρ are the molar mass and the density of W, respectively. The numerator in Equation (6) represents the energy provided by the atoms in the surface layer of the material; the denominator represents the mole number of atoms in the surface layer. Correspondingly, the selection of each variable is shown in Figure 2.

As shown in Figure 2, when the thicknesses of the surface layer ($l_{0}$) are set to different values, the volume of the surface layer ($\Delta V=S\times l_{0}$) would vary, leading to a change in the mole number of atoms in the surface layer (=ρ⋅ΔV/M). Hence, the computed surface energy value ($E_{\mathrm{surf}(M)}$) cannot be stabilized. It is clear that the problem present in the first-principles calculation of surface energy, where the calculated surface energies are dependent on the number of atomic layers, also exists in non-first-principles average molar surface energy calculations due to the positive correlation between the thickness ($l_{0}$) and the number of atomic layers (*t*). Therefore, it is necessary first to define the range of the surface layer when calculations for surface energy in units of kJ/mol are performed.

### 2.4. Determining the Number of Atomic Layers in the Surface Layer

To calculate reasonable surface energies using Equation (4), it is necessary to determine the total number of atoms, $n_{\mathrm{slab}}^{hkl}$ (or the total mole number, $N^{hkl}$), in the surface slab model selected in the computation process. Here, $n_{\mathrm{slab}}^{hkl}$ represents the product of the number of atoms in a monolayer and the number of atomic layers *t* in the surface slab model. When calculating surface energy in units of kJ/mol, it is essential first to determine the appropriate *t* value, i.e., to define the range of atoms belonging to the surface layer in the model. The reliability of calculated surface energies measured in kJ/mol would be ensured only when the model is refined to contain the surface layer atoms exclusively. In previous studies, the convergence tests of surface energy calculations based on first-principles methods were carried out by increasing the number of atomic layers within the slab model and computing the corresponding surface energy value. When the number of atomic layers reaches a specific value, $N_{c}$, a further increase in the number of atomic layers would result in a negligible change in surface energy. Thus, the atoms in the central layers could be regarded as bulk atoms, while $N_{c}$ would be treated as the surface layer thickness of both upper and lower surfaces. However, computational results indicate that when calculating surface energies for specific crystal planes, such as the W(222) plane, the surface energy value would fluctuate with increasing atomic layers in the slab model, leading to difficulty in stable convergence as well as determination of the number of atomic layers in the surface layer. Moreover, due to the significant computational resources required for first-principles calculations, they are generally applicable to systems containing tens to hundreds of atoms. Compared to first-principles methods, molecular dynamics computational methods possess broader temporal and spatial scales. Consequently, the surface layer selection method employed in this study demonstrates superior computational efficiency and a broader computational scale. Therefore, the EFS potential was improved to determine the range of these surface layer atoms by molecular dynamics calculations [16]. Calculations of the energy per atom considering different nearest neighbors were then carried out based on the improved EFS potential function. Therefore, the number of atomic layers and the number of atoms in the “surface layer” can be determined from these calculation results. The relevant formulas utilized in this work are outlined below [22,23]:

(7) $$u_{\mathrm{tot}}=\frac{1}{2}\sum_{ij}V(r_{ij})+\sum_{i}f(\rho_{i})$$

$u_{\mathrm{tot}}$, the total energy of a single atom, can be divided into pairwise and n-body terms, then further expanded as Equation (7), expressed as the sum of these two parts. $V(r_{ij})$ represents the pairwise contribution. In this equation, $r_{ij}$ is the distance between atom *i* and atom *j*, while the variable ri represents the local electronic charge density at the *i*-site. The polynomial describing $V(r_{ij})$ in the EFS potential function is extended to a six-order term to optimize the description of interatomic interactions, as shown in Equation (8) [15].

(8) $$V\left(r\right)=\left\{\begin{array}{ll} {\left(r-c\right)}^{2}\left(c_{0}+c_{1}r+c_{2}r^{2}+c_{3}r^{3}+c_{4}r^{4}\right), & r\leq c\\ 0 & r>c \end{array}\right.$$

(9) $$f(\rho_{i})=-A\sqrt{\rho_{i}}$$

(10) $$\rho_{i}=\sum_{j\neq i}\phi \left(r_{ij}\right)$$

(11) $$\phi \left(r\right)=\left\{\begin{array}{ll} {\left(r-d\right)}^{2}+B{\left(r-d\right)}^{4}, & r\leq d\\ 0 & r>d \end{array}\right.$$

In Equation (8), *r* represents the interatomic distance, with $r=r_{ij}$ assumed in the calculations. According to the tight-binding theory, the embedded energy $f(\rho_{i})$ is shown in Equations (9) and (10).

$\phi \left(r_{ij}\right)$ is the single-atom electron density function, with the expression shown in Equation (11). In the equations above, *c* and *d* are cutoff distances, while $c_{0},c_{1},c_{2},c_{3},c_{4},A,B$ are parameters to be fitted. The appropriate values of the nine potential parameters mentioned above are determined by fitting them to physical properties.

Interatomic interactions were only considered up to the third nearest neighbor in previous work [16,24]. In this work, the fourth nearest-neighbor interactions were taken into account and applied to the derivations of the expressions for W physical properties. At this point, it is feasible to derive the potential parameter values above by fitting them to experimental data.

**Figure 3:**
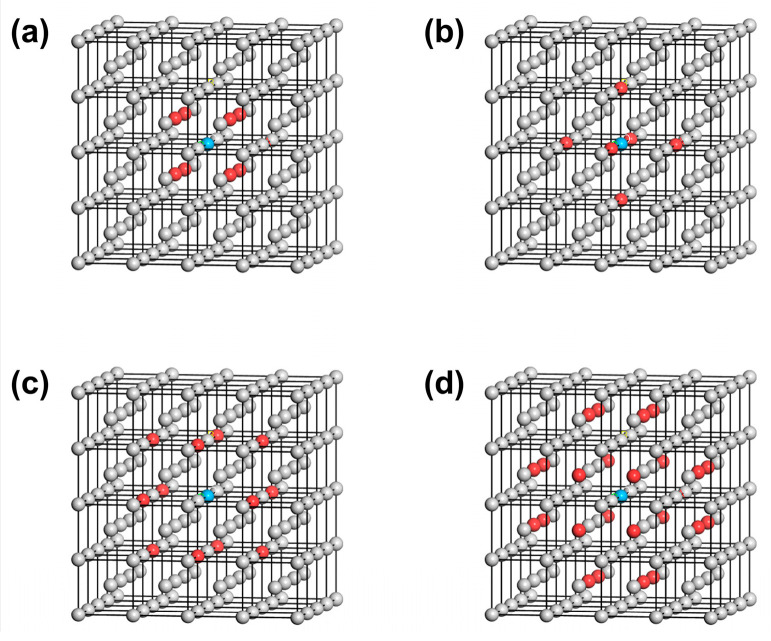
Distribution of the first (**a**), second (**b**), third (**c**), and fourth (**d**) nearest neighbor atoms of W metal. The reference atom is colored blue, while the nearest neighbor atoms are colored red.

First, the distribution of the first to fourth nearest neighbor atoms of the reference atom (selecting the atom at the origin $\begin{array}{l} \left(\begin{array}{l} 0,0,0 \end{array}\right) \end{array}$) in the BCC W is shown in Figure 3. There are eight atoms in the first nearest neighbor, with atomic coordinates $\begin{array}{l} \left(\begin{array}{l} \frac{1}{2}a,\frac{1}{2}a,\pm \frac{1}{2}a \end{array}\right) \end{array}$, $\begin{array}{l} \left(\begin{array}{l} -\frac{1}{2}a,\frac{1}{2}a,\pm \frac{1}{2}a \end{array}\right) \end{array}$, $\begin{array}{l} \left(\begin{array}{l} -\frac{1}{2}a,-\frac{1}{2}a,\pm \frac{1}{2}a \end{array}\right) \end{array}$, and $\begin{array}{l} \left(\begin{array}{l} \frac{1}{2}a,-\frac{1}{2}a,\pm \frac{1}{2}a \end{array}\right) \end{array}$. 6 atoms are located in the second nearest neighbor with atomic coordinates of $\begin{array}{l} \left(\begin{array}{l} 0,0,\pm a \end{array}\right) \end{array}$, $\begin{array}{l} \left(\begin{array}{l} 0,\pm a,0 \end{array}\right) \end{array}$ and $\begin{array}{l} \left(\begin{array}{l} \pm a,0,0 \end{array}\right) \end{array}$. The third nearest neighbor contains 12 atoms with atomic coordinates $\begin{array}{l} \left(a,0,\pm a\right) \end{array}$, $\begin{array}{l} \left(-a,0,\pm a\right) \end{array}$, $\begin{array}{l} \left(0,a,\pm a\right) \end{array}$, $\begin{array}{l} \left(0,-a,\pm a\right) \end{array}$, $\begin{array}{l} \left(a,\pm a,0\right) \end{array}$ and $\begin{array}{l} \left(-a,\pm a,0\right) \end{array}$. Also, there are 24 atoms included in the fourth nearest neighbor of the reference atom, the atomic coordinates of which are $\begin{array}{l} \left(\begin{array}{l} \frac{1}{2}a,\frac{1}{2}a,\pm \frac{3}{2}a \end{array}\right) \end{array}$, $\begin{array}{l} \left(\begin{array}{l} \frac{1}{2}a,-\frac{1}{2}a,\pm \frac{3}{2}a \end{array}\right) \end{array}$, $\begin{array}{l} \left(\begin{array}{l} -\frac{1}{2}a,\frac{1}{2}a,\pm \frac{3}{2}a \end{array}\right) \end{array}$, $\begin{array}{l} \left(\begin{array}{l} -\frac{1}{2}a,-\frac{1}{2}a,\pm \frac{3}{2}a \end{array}\right) \end{array}$, $\left(\frac{1}{2}a,\frac{3}{2}a,\pm \frac{1}{2}a\right)$, $\left(-\frac{1}{2}a,\frac{3}{2}a,\pm \frac{1}{2}a\right)$, $\left(-\frac{1}{2}a,-\frac{3}{2}a,\pm \frac{1}{2}a\right)$, $\left(\frac{1}{2}a,-\frac{3}{2}a,\pm \frac{1}{2}a\right)$, $\left(\frac{3}{2}a,\frac{1}{2}a,\pm \frac{1}{2}a\right)$, $\left(-\frac{3}{2}a,\frac{1}{2}a,\pm \frac{1}{2}a\right)$, $\left(-\frac{3}{2}a,-\frac{1}{2}a,\pm \frac{1}{2}a\right)$ and $\left(\frac{3}{2}a,-\frac{1}{2}a,\pm \frac{1}{2}a\right)$. Correspondingly, the first to fourth nearest-neighbor distances satisfy $r_{1}=\frac{\sqrt{3}}{2}a$, $r_{2}=a$, $r_{3}=\sqrt{2}a$ and $r_{4}=\frac{\sqrt{11}}{2}a$, where *a* = 3.165 Å corresponds to the lattice constant of W.

According to Equation (12), the energy change of a single W atom before and after cleavage along the (*hkl*) crystal plane can be calculated as follows.

(12) $$\Delta u_{s}^{hkl}(k)=C\cdot N_{A}\cdot \left[\sum_{v=1}^{v^{\prime }}\Delta u_{v}^{hkl}(k)\right](v=1,2,\cdot \cdot \cdot v^{\prime })$$

$\Delta u_{v}^{hkl}(k)$ represents the change in total energy ($u_{\mathrm{tot}}$) per atom before and after cleavage along the *v*-th (*hkl*) layer, considering the interactions between the first to *k*-th nearest neighbor atoms, calculated from Equation (8), noting that the total energies per atom before and after cleavage are $u_{\mathrm{tot},0}(k)$ and $u_{\mathrm{tot},1}^{hkl}(k)$, respectively.

(13) $$\Delta u_{v}^{hkl}(k)=u_{\mathrm{tot},1}^{hkl}(k)-u_{\mathrm{tot},0}\left(k\right)$$

This energy change $\Delta u_{v}^{hkl}(k)$, which originates from the coordination number change of atoms before and after cleavage, is equivalent to the energy difference ($\Delta E^{hkl}$) between the energies of the (*hkl*) surface slab model ($E_{\mathrm{slab}}^{hkl}$) and the bulk model ($E_{\mathrm{bulk}}$) in the surface energy calculation formula (Equation (2)), where $\Delta E^{hkl}=E_{\mathrm{slab}}^{hkl}-n^{hkl}\cdot E_{\mathrm{bulk}}$ and $n^{hkl}$ represents the ratio of the number of atoms in the surface slab model to that in the bulk model. The value of $\Delta u_{v}^{hkl}(k)$ is affected by the nearest neighbor atoms considered in the calculation process. For example, when only the interatomic interactions from the first to fourth nearest neighbor atoms are considered, the atoms in this range would no longer be changed by cleavage once the distance from the reference atom to the cleavage surface exceeds the fourth nearest-neighbor distance. Accordingly, the energy change induced by this cleavage operation equals zero, i.e., $\Delta u_{v}^{hkl}(k)$ = 0.

It can be concluded from the definition provided that using different nearest-neighbor distances in calculations will lead to different energy changes ($\Delta u_{s}$). The more distant the nearest neighbor the atoms belong to, the weaker the corresponding interactions would be. Theoretically, there exists a specific number of nearest neighbors, called $k_{0}$, where the difference in the value of $\Delta u_{s}(k_{0})$—considering interactions up to the $k_{0}$-th nearest neighbor—and the value of $\Delta u_{s}(k_{0}+1)$, which includes interactions up to the $\left(k_{0}+1\right)$-th nearest neighbor, becomes negligible. This indicates that the interatomic interactions of the $\left(k_{0}+1\right)$-th nearest neighbor atoms are insignificant for the calculations. Once $k_{0}$ is identified, the delineation between the surface layer atoms and the internal atoms can be determined. Correspondingly, the number of atomic layers in the surface layer for calculating surface energies (in kJ/mol) will be designated as $t_{0}$. As a result, this work focuses on the interatomic interactions within the first to fourth nearest-neighbor atoms, with the cutoff distance defined between the fourth and fifth nearest-neighbor distances.

The number of nearest-neighbor atoms would change in various magnitudes by selecting different cleavage planes. Therefore, the energy change per atom before and after cleaving along the (*hkl*) crystal plane (considering the first to fourth nearest neighbor) is also different. Taking W(110) as an example, the energy change per atom before and after cleavage is noted as $\Delta u_{s}^{110}(4)$. The formula for $\Delta u_{s}^{110}(4)$ is derived as follows.

**Figure 4:**
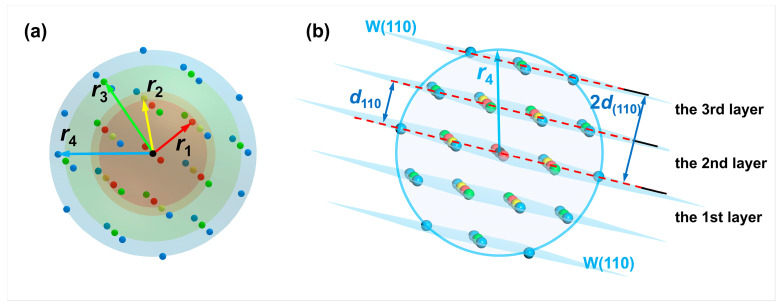
Schematics of the reference atom, nearest-neighbor atoms, and crystal planes formed by cleavage. (**a**) The distribution of the first to fourth nearest neighbor atoms of an arbitrary atom in the perfect W crystal. The reference atom is black-colored. The first to fourth nearest-neighbor distances are noted as *r*_1_, *r*_2_, *r*_3_, and *r*_4_, respectively; (**b**) schematic of the reference atom (center of the sphere) and the parallel W(110) crystal planes consisting of the first to fourth nearest neighbor atoms (*d*_110_ is the interplanar spacing of W(110) crystal plane.

The reference atom can be arbitrary in the bulk phase. The first to fourth nearest neighbor atoms (colored in red, yellow, green, and blue) of the reference atom (colored in black) in the perfect crystal arrangement of W are illustrated in Figure 4a. In the meantime, the W(110) crystal plane where the reference atom is located can be set as the first W(110) layer. Since the interplanar spacing of the W(110) crystal plane is $d_{110}$ = 2.2380 Å, there is also a distribution of atoms in parallel planes at distances from the first W(110) layer that are integer multiples of $d_{110}$, such as $2d_{110}$, $3d_{110}$, and so on. The parallel plane at a distance $d_{110}$ = 2.2380 Å from the first layer is set to the second W(110) layer for research purposes. Similarly, the third layer is set at a distance $2d_{110}$ = 4.4760 Å from the first W(110) layer, as illustrated in Figure 4b.

A sphere is created with the reference atom as the center and the fourth nearest-neighbor distance as the radius, as depicted in Figure 4b. A maximum of two layers of the W(110) crystal planes on each side of the first W(110) plane, where the reference atom is located, intersect with the sphere, indicating that the number of atoms in the fourth nearest neighbor range would change when cleaving along the first to third W(110) layers. On the contrary, the number of these atoms would no longer change when the cleavage operation exceeds the fourth nearest-neighbor distance from the reference atom.

**Figure 5:**
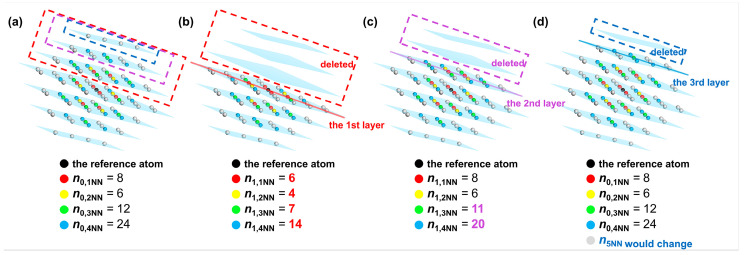
Distribution of the first to fifth nearest neighbor atoms of the reference atom in the perfect W crystal before cleavage (**a**) and after cleaving along the (**b**) first, (**c**) second and (**d**) third W(110) layers. The W(110) layers are colored blue. The first to fifth nearest neighbor atoms are colored red, yellow, green, blue, and gray, respectively.

According to Equations (12) and (13), the energy change induced by the cleavage operation considering interatomic interactions between the reference atom and its first to fourth nearest neighbor atoms is equal to the sum of the energy change when cleavage is carried out along the first to the third W(110) crystal planes for a single W atom, which is deduced as follows. Variable $n_{0,k\mathrm{NN}}$ represents the number of the *k*-th nearest neighbor atoms to the reference atom before cleavage. In contrast, those *k*-th nearest neighbor atoms after cleavage are denoted as variable $n_{1,k\mathrm{NN}}$. When cleavage is carried out along the first W(110) layer, the atoms located in the first to fourth nearest neighbors to the reference atom exhibit the change illustrated in Figure 5a to Figure 5b. It can be shown that the number of the first nearest neighbor atoms (colored red) changes from 8 to 6, and the number of second nearest neighbor atoms (colored yellow) changes from 6 to 4. Meanwhile, the number of third and fourth nearest neighbor atoms (colored green and blue) changes from 12 to 7 and from 24 to 14, respectively. Similarly, when the cleavage is applied along the second and third W(110) layer, the first to fourth nearest-neighbor atoms are changed from the initial distribution in Figure 5a to those shown in Figure 5c,d, respectively. The grey atoms represent atoms situated more than the fourth nearest-neighbor distance from the reference atom. Since the cutoff distance of the interatomic interactions is set between the fourth and fifth nearest-neighbor distances, the cohesive and repulsive interactions of the first to fourth nearest neighbors are included in calculations of the energy per atom. The interactions of the fifth and higher nearest neighbor atoms are not considered in this analysis.

Based on the difference in the number of the first to fourth nearest-neighbor atoms before and after cleavage, along with Equations (7)–(11), the values of the total energy per atom before and after cleavage ($u_{\mathrm{tot},0}(4)$ and $u_{\mathrm{tot},1}^{hkl}(4)$) can be calculated. “(4)” indicates that atoms up to the fourth nearest neighbor were selected in the computation. Afterwards, the difference between the total energy $u_{\mathrm{tot},1}^{110}(4)$ and $u_{\mathrm{tot},0}(4)$ before and after cleavage along the first to *v*′-th W(110) crystal plane can be obtained based on Equations (12) and (13). Finally, the energy difference before and after cleavage along each layer was added up to $\sum_{v=1}^{v^{\prime }}\Delta u_{v}^{110}(4)$ and expressed in units of eV. As shown in Figure 5, the value of *v*′ satisfying $\Delta u_{v}^{110}(4)\neq 0$ equals 2. The calculation process and result are shown in Equation (14). The energy change per atom before and after cleavage along the W(110) crystal plane, accounting for interatomic interactions within the fourth nearest-neighbor range in units of kJ/mol, denoted as $\Delta u_{s}^{110}(4)$, can be obtained by dividing $\sum_{v=1}^{2}\Delta u_{v}^{110}(4)$ by the mole number of a single atom and adjusting units via the conversion factor according to Equation (15). It should be mentioned that the values of terms $V(r_{ij})$ and $\phi \left(r_{ij}\right)$ in Equation (7) are abbreviated for convenience for different $r_{ij}$ values, when the interatomic interactions are in the first nearest neighbor, $V_{1}=V\left(\frac{\sqrt{3}}{2}a\right)$ and $\phi_{1}=\phi \left(\frac{\sqrt{3}}{2}a\right)$. By analogy, it can be inferred that $V_{4}=V\left(\frac{\sqrt{11}}{2}a\right)$ and $\phi_{4}=\phi \left(\frac{\sqrt{11}}{2}a\right)$ for interatomic interactions in the fourth nearest neighbor.

(14) $$\begin{array}{ll} \sum_{v=1}^{2}\Delta u_{v}^{110}(4) & =\sum_{v=1}^{2}\left[u_{\mathrm{tot},1}^{110}(4)-u_{\mathrm{tot},0}(4)\right]\\ & =\left[\frac{1}{2}\left(6V_{1}+4V_{2}+7V_{3}+14V_{4}\right)-A\sqrt{6\varphi_{1}+4\varphi_{2}+7\varphi_{3}+14\varphi_{4}}\right]\\ & +\left[\frac{1}{2}\left(8V_{1}+6V_{2}+11V_{3}+20V_{4}\right)-A\sqrt{8\varphi_{1}+6\varphi_{2}+11\varphi_{3}+20\varphi_{4}}\right]\\ & -2\left[\frac{1}{2}\left(8V_{1}+6V_{2}+12V_{3}+24V_{4}\right)-A\sqrt{8\varphi_{1}+6\varphi_{2}+12\varphi_{3}+24\varphi_{4}}\right]\\ & =-V_{1}-V_{2}-3V_{3}-7V_{4}+2A\sqrt{8\varphi_{1}+6\varphi_{2}+12\varphi_{3}+24\varphi_{4}}\\ & -A\sqrt{6\varphi_{1}+4\varphi_{2}+7\varphi_{3}+14\varphi_{4}}-A\sqrt{8\varphi_{1}+6\varphi_{2}+11\varphi_{3}+20\varphi_{4}} \end{array}$$

(15) $$\begin{array}{ll} \Delta u_{s}^{110}\left(4\right) & =CN_{A}\sum_{v=1}^{2}\Delta u_{v}^{110}(4)\\ & =CN_{A}\left(-V_{1}-V_{2}-3V_{3}-7V_{4}+2A\sqrt{8\varphi_{1}+6\varphi_{2}+12\varphi_{3}+24\varphi_{4}}\right.\\ & \left.-A\sqrt{6\varphi_{1}+4\varphi_{2}+7\varphi_{3}+14\varphi_{4}}-A\sqrt{8\varphi_{1}+6\varphi_{2}+11\varphi_{3}+20\varphi_{4}}\right) \end{array}$$

(16) $$\begin{array}{ll} \Delta u_{s}^{200}\left(4\right) & =CN_{A}\left(-2V_{1}-V_{2}-4V_{3}-10V_{4}+3A\sqrt{8\varphi_{1}+6\varphi_{2}+12\varphi_{3}+24\varphi_{4}}\right.\\ & -A\sqrt{4\varphi_{1}+5\varphi_{2}+8\varphi_{3}+12\varphi_{4}}-A\sqrt{8\varphi_{1}+5\varphi_{2}+8\varphi_{3}+20\varphi_{4}}\\ & \left.-A\sqrt{8\varphi_{1}+6\varphi_{2}+12\varphi_{3}+20\varphi_{4}}\right) \end{array}$$

(17) $$\begin{array}{ll} \Delta u_{s}^{222}\left(4\right) & =CN_{A}\left(-3V_{1}-3V_{2}-6V_{3}-18V_{4}+5A\sqrt{8\varphi_{1}+6\varphi_{2}+12\varphi_{3}+24\varphi_{4}}\right.\\ & -A\sqrt{4\varphi_{1}+3\varphi_{2}+9\varphi_{3}+12\varphi_{4}}-A\sqrt{7\varphi_{1}+3\varphi_{2}+9\varphi_{3}+15\varphi_{4}}\\ & -A\sqrt{7\varphi_{1}+6\varphi_{2}+9\varphi_{3}+15\varphi_{4}}-A\sqrt{8\varphi_{1}+6\varphi_{2}+9\varphi_{3}+21\varphi_{4}}\\ & \left.-A\sqrt{8\varphi_{1}+6\varphi_{2}+12\varphi_{3}+21\varphi_{4}}\right) \end{array}$$

$\Delta u_{s}^{200}(4)$ and $\Delta u_{s}^{222}(4)$, which represent the energy change of a single atom before and after cleaving along the W(200) and W(222) crystal planes, considering the first to fourth nearest-neighbor interatomic interactions, can be calculated similarly according to Equations (16) and (17).

When calculations are applied, $V_{4}$ and $\phi_{4}$ are set to zero, considering the first to third nearest-neighbor interatomic interactions. At this time, the energy change of a single atom before and after cleaving along the W(110) crystal plane can be calculated with Equation (18), which is modified from Equation (15).

(18) $$\begin{array}{ll} \Delta u_{s}^{110}\left(3\right) & =CN_{A}\left(-V_{1}-V_{2}-3V_{3}+2A\sqrt{8\varphi_{1}+6\varphi_{2}+12\varphi_{3}}\right.\\ & \left.-A\sqrt{6\varphi_{1}+4\varphi_{2}+7\varphi_{3}}-A\sqrt{8\varphi_{1}+6\varphi_{2}+11\varphi_{3}}\right) \end{array}$$

Equation (15) is converted into Equation (19) when only the first and second nearest-neighbor interatomic interactions are considered. Likewise, the energy change calculations of W(200) and W(222) can be converted using this method.

(19) $$\Delta u_{s}^{110}\left(2\right)=CN_{A}\left(-V_{1}-V_{2}+A\sqrt{8\varphi_{1}+6\varphi_{2}}\right.\left.-A\sqrt{6\varphi_{1}+4\varphi_{2}}\right)$$

Fitting the physical properties of W by the least-squares method based on the improved potential function would facilitate obtaining suitable values of the nine potential parameters ($c,d,c_{0},c_{1},c_{2},c_{3},c_{4},A,B$). The fitted quantities include pressure (P), bulk elastic modulus ($B_{m}$), shear elastic modulus ($C_{p}$), and elastic constants ($C_{44}$). The defining formula of P [22] and the derived expressions of $B_{m}$, $C_{p}$, and $C_{44}$ of BCC W based on the improved EFS potential are shown in Equations (20)–(23). Meanwhile, molecular dynamics calculations were applied in this work to determine the lattice constant (a), the cohesive energy ($E_{\mathrm{coh}}$), and the vacancy formation energy ($E_{\mathrm{vac}}$) based on the modified EFS potential function. Then, the reliability of the obtained potential parameters was confirmed by comparing these calculated values and experimental values.

(20) $$P=-\frac{du_{\mathrm{tot}}}{d\Omega }$$

(21) $$\begin{array}{ll} B_{m} & =-\Omega \frac{dP}{d\Omega }\\ & =-\frac{2}{9a^{2}}\left[4\sqrt{3}\right.{V_{1}}^{\prime }+6{V_{2}}^{\prime }+12\sqrt{2}{V_{3}}^{\prime }+12\sqrt{11}{V_{4}}^{\prime }-3a{V_{1}}^{\prime \prime }-3a{V_{2}}^{\prime \prime }-12a{V_{3}}^{\prime \prime }-33a{V_{4}}^{\prime \prime }\\ & +f^{\prime }(\rho )\left(8\sqrt{3}{\phi_{1}}^{\prime }+12{\phi_{2}}^{\prime }+24\sqrt{2}{\phi_{3}}^{\prime }+24\sqrt{11}{\phi_{4}}^{\prime }-6a{\phi_{1}}^{\prime \prime }-6a{\phi_{2}}^{\prime \prime }-24a{\phi_{3}}^{\prime \prime }-66a{\phi_{4}}^{\prime \prime }\right)\\ & \left.-4af^{\prime \prime }(\rho ){\left(2\sqrt{3}{\phi_{1}}^{\prime }+3{\phi_{2}}^{\prime }+6\sqrt{2}{\phi_{3}}^{\prime }+6\sqrt{11}{\phi_{4}}^{\prime }\right)}^{2}\right] \end{array}$$

(22) $$\begin{array}{ll} C_{P} & =C_{P,P}+C_{P,M}=\frac{1}{4}\cdot \frac{\partial^{2}W_{P}}{\partial \varepsilon_{11}^{2}}+\frac{1}{4}\cdot \frac{\partial^{2}W_{M}}{\partial \varepsilon_{11}^{2}}\\ & =\frac{1}{a^{2}}\left[\frac{4\sqrt{3}}{3}{V_{1}}^{\prime }+a{V_{2}}^{\prime \prime }+{V_{2}}^{\prime }+a{V_{3}}^{\prime \prime }+\frac{7\sqrt{2}}{2}{V_{3}}^{\prime }+\frac{64}{11}a{V_{4}}^{\prime \prime }+\frac{356\sqrt{11}}{121}{V_{4}}^{\prime }\right.\\ & \left.+f^{\prime }\left(\rho \right)\left(\frac{8\sqrt{3}}{3}{\phi_{1}}^{\prime }+2a{\phi_{2}}^{\prime \prime }+2{\phi_{2}}^{\prime }+2a{\phi_{3}}^{\prime \prime }+7\sqrt{2}{\phi_{3}}^{\prime }+\frac{128}{11}a{\phi_{4}}^{\prime \prime }+\frac{712\sqrt{11}}{121}{\phi_{4}}^{\prime }\right)\right] \end{array}$$

(23) $$\begin{array}{ll} C_{44} & =C_{44,P}+C_{44,M}=\frac{1}{4}\cdot \frac{\partial^{2}W_{P}}{\partial \varepsilon_{12}^{2}}+\frac{1}{4}\cdot \frac{\partial^{2}W_{M}}{\partial \varepsilon_{12}^{2}}\\ & =\frac{1}{a^{2}}\left[\frac{2}{3}a{V_{1}}^{\prime \prime }+\frac{8\sqrt{3}}{9}{V_{1}}^{\prime }+2{V_{2}}^{\prime }+2a{V_{3}}^{\prime \prime }+3\sqrt{2}{V_{3}}^{\prime }+\frac{38}{11}a{V_{4}}^{\prime \prime }+\frac{408\sqrt{11}}{121}{V_{4}}^{\prime }\right.\\ & \left.+f^{\prime }\left(\rho \right)\left(\frac{4}{3}a{\phi_{1}}^{\prime \prime }+\frac{16\sqrt{3}}{9}{\phi_{1}}^{\prime }+4{\phi_{2}}^{\prime }+4a{\phi_{3}}^{\prime \prime }+6\sqrt{2}{\phi_{3}}^{\prime }+\frac{76}{11}a{\phi_{4}}^{\prime \prime }+\frac{816\sqrt{11}}{121}{\phi_{4}}^{\prime }\right)\right] \end{array}$$

In the equations above, Ω stands for the volume corresponding to a single atom. ${V_{i}}^{\prime }$, ${\phi_{i}}^{\prime }$ and $f^{\prime }(\rho )$ are the first-order derivative terms, while ${V_{i}}^{\prime \prime }$, ${\phi_{i}}^{\prime \prime }$ and $f^{\prime \prime }(\rho )$ are the second-order derivative terms, respectively. W represents the strain energy density, while $\varepsilon_{11}$ and $\varepsilon_{12}$ are the strain components. The components denoted by P and M in the derivation process represent the contributions of pairwise and n-body parts, respectively.

The energy change with different nearest neighbor atoms considered before and after cleavage along different low-index (*hkl*) crystal planes (noted as $\Delta u_{s}^{hkl}(k)$, *k* = 2,3,4) can be calculated using the optimal potential parameters obtained by the fitting operation. Meanwhile, the differences in $\Delta u_{s}^{hkl}(k)$ values considering interatomic interactions of different nearest neighbors were calculated by Equations (24) and (25), and then the $\left|\Delta_{k,k+1}\left(u_{s}^{hkl}\right)\right|$ value, which is negligible enough, can be determined. Based on the calculation result, the *k*-th nearest neighbor can be determined to meet the conditions, and furthermore, the thickness and the number of atomic layers in the surface layer of the surface slab model can be derived for their application in the computation of the first-principles surface energies measured in kJ/mol for this model.

(24) $$\Delta_{23}\left(u_{s}^{hkl}\right)=\left[\Delta u_{s}^{hkl}\left(2\right)-\Delta u_{s}^{hkl}\left(3\right)\right]/\Delta u_{s}^{hkl}\left(3\right)$$

(25) $$\Delta_{34}\left(u_{s}^{hkl}\right)=\left[\Delta u_{s}^{hkl}\left(3\right)-\Delta u_{s}^{hkl}\left(4\right)\right]/\Delta u_{s}^{hkl}\left(4\right)$$

## 3. Results and Discussion

### 3.1. The Morphology and Electrochemical Activity of Nanoporous Structure

Firstly, nanoporous layers were synthesized to investigate the influence of nano-treatment on the surface properties of W, which was subsequently applied in surface energy calculations, providing experimental validation for the calculation method. In this work, a novel nanosized method based on anodization and deoxidation annealing was applied to obtain a nanoporous structure on the W surface.

**Figure 6:**
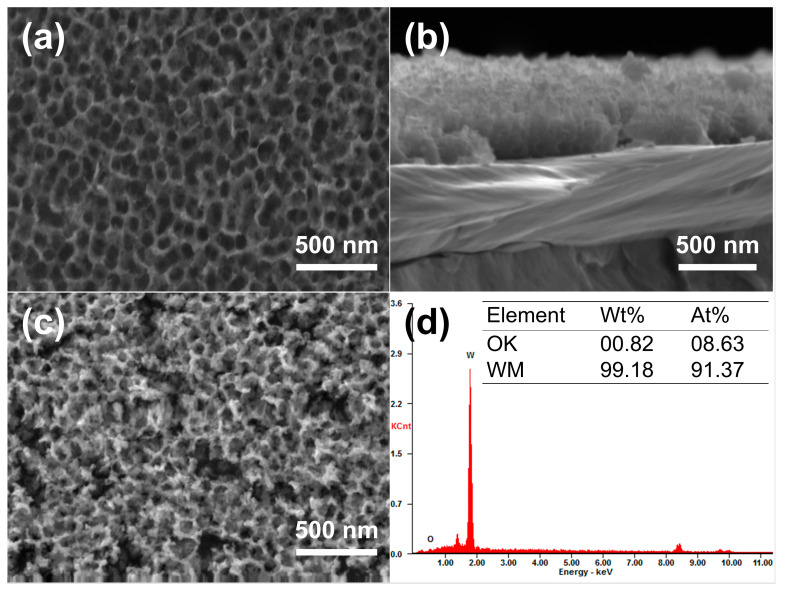
Morphologies and chemical compositions of the nanoporous W layer. (**a**) Surface and (**b**) cross-section morphologies of the nanoporous W layer after anodization; (**c**) surface morphology and (**d**) corresponding EDS results of the nanoporous layer after deoxidation annealing at 600 °C.

Anodization was performed in an electrolyte containing 0.5 wt% NaF (mass fraction) and 1 mol/L Na_2_SO_4_ [17]. Since anodization parameters would influence the formation and dissolution rates of WO*_x_* (*x* = 2.72–2.90), and consequently the resulting morphology of the nanoporous structure, the anodizing voltage, time, and temperature were adjusted to achieve the best match of parameters and, ultimately, to obtain the optimal morphology of the nanopores in this experiment. The optimum anodized nanoporous layer was obtained under the anodizing condition of 50 V, 20 °C, and 7 min, consisting of uniformly distributed nanopores with an average pore size of about 130 nm and a depth of up to 200 nm, as depicted in Figure 6a,b. Since the nanoporous tungsten layer obtained by anodization mainly consists of W and tungsten oxide (W and WO*_x_*), deoxidation annealing of the anodized samples was carried out to remove the oxygen. It was verified by experiments that selecting appropriate annealing parameters is necessary for the deoxidation annealing process. After constant temperature annealing at 600 °C for 3 h in a hydrogen atmosphere, the surface morphology and the corresponding EDS results of the nanoporous layer on the W surface are shown in Figure 6c,d. As shown in Figure 6c, there were no significant changes observed in the overall morphology of the nanoporous layer. Moreover, the table displayed in Figure 6d lists the contents of tungsten and oxygen in the porous layer after deoxidation annealing. The results indicate that the oxygen content in the porous layer after annealing was 0.82 wt.%, suggesting that the oxygen element could be removed with the nanoporous structure maximally retained. Apparently, nanoporous layers of pure tungsten were successfully synthesized on the W surface in this work.

**Figure 7:**
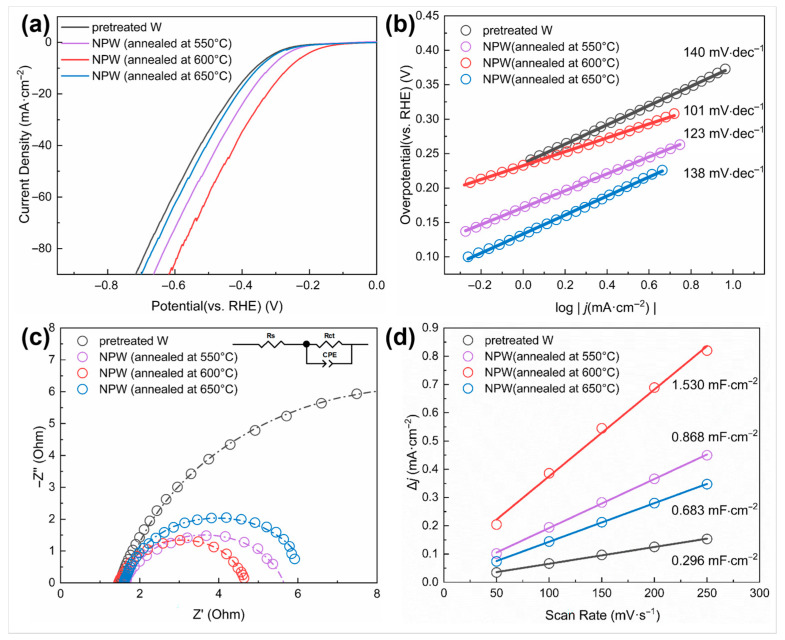
Electrochemical properties of W samples before and after nano-treatment under different deoxidation annealing conditions. (**a**) Polarization curves and (**b**) the corresponding Tafel plot; (**c**) Nyquist plots of the W samples under the potential of −0.30 V (vs. RHE) (Inset is the equivalent circuit model; $R_{\mathrm{ct}}$—charge transfer resistance, $R_{s}$—Ohmic series resistance, CPE—constant phase element); (**d**) plot of capacitive current density as a function of scan rate to compare double-layer capacitance ($C_{\mathrm{dl}}$).

The effect of nano-treatment on surface activity was measured by electrochemical tests and comparison between the pretreated W samples, which were ground and polished only, and those NPW foils annealed at different temperatures. Figure 7a shows the linear sweep voltammetry (LSV) curves of the W samples. It can be seen that the NPW (annealed at 600 °C) exhibited the lowest overpotential ($\eta_{10}$ = 262 mV) when the current density reached 10 mA/cm^2^, which was lower than W without nano-treatment (362 mV), and NPW deoxidized at 550 °C (318 mV) and 650 °C (352 mV). Moreover, the $\eta_{1}$ of nano-treated W (annealed at 600 °C) was 124 mV, lower than that of W without nano-treatment (211 mV), indicating higher surface activity. According to the Tafel plot converted from the LSV polarization curves (Figure 7b), the Tafel slope of NPW annealed at 600 °C (101 mV/dec) was much lower than that of W without nano-treatment (140 mV/dec), indicating that nano-treated W exhibited superior reaction kinetics of HER. In addition, the Nyquist plot represents the impedance information at −0.56 V versus (vs.) SCE, i.e., −0.3 V vs. RHE, as shown in Figure 7c, from the electrochemical impedance spectroscopy (EIS) technique employed. Compared to W without nano-treatment, NPW annealed at 600 °C had a smaller-diameter semicircle in the low-frequency range of the Nyquist plot, which means that it had a smaller value of $R_{\mathrm{ct}}$ (3.162 Ohm compared to 15.04 Ohm), a faster rate of HER charge transfer, and better electrochemical performance. Moreover, A linear fit is applied and demonstrated in Figure 7d, with capacitive current densities as the dependent variable and scan rates of cyclic voltammetry (CV) tests as the independent variable (varying from 50 to 250 mV/s in this work). The obtained slope value would correspond to $C_{\mathrm{dl}}$. It can be seen from Figure 7d that the estimated $C_{\mathrm{dl}}$ value of NPW (600 °C annealed) is much higher than that of W without nano-treatment, indicating that nano-treatment led to a larger effective electrochemical active surface area since the electrochemical active surface area (ECSA) could be considered proportional to the double-layer capacitance ($C_{\mathrm{dl}}$) [25]. Since the nanoporous tungsten produced in this study is intended for use as a disposable composite interconnect material, rather than as an electrocatalytic or battery material, long-term stability tests on the tungsten samples were not conducted.

The above test results indicate an increase in surface activity after nano-treatment. Furthermore, the 600 °C-annealed W samples exhibit optimal properties, confirming that the novel nanosized method adopted in this work would effectively enhance the surface activity of W. The reason for the enhancement in surface activity will be further explored through the calculations below.

### 3.2. Calculation of Surface Energy in Different Units of Measurement

Nano-treatment would lead to enhanced activity for several reasons, among which the increase in surface energy is an essential origin [7,10]. Moreover, it has been observed that various crystal planes possess different surface energy values [26]. The increase in surface energy is likely caused by the rise in the ratio of high-surface-energy crystal planes, which could affect surface activity.

Since the surface energy of a specific W crystal plane is challenging to determine by experiment, first-principles calculations were adopted to calculate the surface energy of various crystal planes ($E_{\mathrm{surf}}^{hkl}$) using the CASTEP module in the Materials Studio software. In addition to computing surface energies measured in J/m^2^, we proposed a calculation method for surface energies measured in kJ/mol from the perspective of applications in thermodynamic calculations. Therefore, it is possible to make a semi-quantitative comparison of the effect of nano-treatment on the total surface energy of W by analyzing the change in content of crystal planes and surface energy values of each crystal plane using this method.

#### 3.2.1. Calculation of Surface Energies in J/m^2^

**Figure 8:**
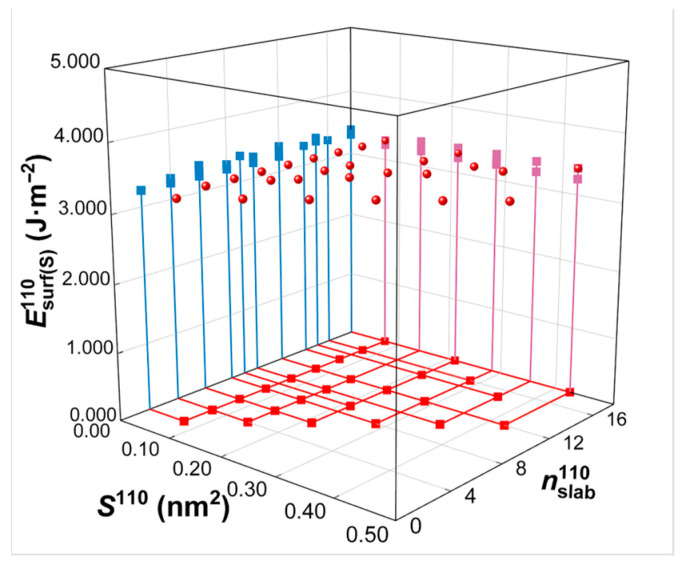
Surface energy calculation in units of J/m^2^. $E_{\mathrm{surf}(s)}^{110}$ represents the surface energy for variations in surface area ($S^{110}$) and total number of atoms ($n_{\mathrm{slab}}^{110}$) according to the surface slab model of the W(110) crystal plane. The projection of the three-dimensional coordinates $\left(S^{110},n_{\mathrm{slab}}^{110},E_{\mathrm{surf}(s)}^{110}\right)$ onto the three coordinate planes is illustrated in different colors.

Calculated from Equations (2) and (3), the commonly used first-principles method for surface energy calculations, the resulting surface energy values measured in J/m^2^ are shown in Table 1. In actual surface energy calculations, instead of selecting default values, $S^{hkl}$, the surface area in the model, and $n_{\mathrm{slab}}^{hkl}$, the total number of atoms in the model, are manually selected during the construction process of the model to ensure accuracy and usability. The surface energy values calculated from Equation (2) for the same crystal plane (e.g., W(110) crystal plane) with various $S^{110}$ and $n_{\mathrm{slab}}^{110}$ values chosen for model construction are demonstrated in Figure 8. These results indicate that the surface energies in J/m^2^ remain nearly constant, with minor fluctuations of less than 5%, regardless of the variation in $S^{hkl}$ and $n_{\mathrm{slab}}^{hkl}$.

#### 3.2.2. Calculation of Surface Energies in kJ/mol

In contrast to the surface energy measured in J/m^2^ ($E_{\mathrm{surf}(s)}^{hkl}$), the value of surface energy measured in kJ/mol ($E_{\mathrm{surf}(M)}^{hkl}$), calculated by Equation (4), is greatly influenced by the selected $S^{hkl}$ and $n_{\mathrm{slab}}^{hkl}$ values. $E_{\mathrm{surf}(M)}^{hkl}$ cannot be stabilized around a specific value when different $S^{hkl}$ and $n_{\mathrm{slab}}^{hkl}$ values are selected. Therefore, it is essential to identify the factors affecting these values before selecting the appropriate value of surface energies measured in kJ/mol.

(26) $$E_{\mathrm{surf}(M)}^{hkl}[\mathrm{kJ}\cdot {\mathrm{mol}}^{-1}]=K\cdot \frac{S^{hkl}}{n_{\mathrm{slab}}^{hkl}}$$

**Figure 9:**
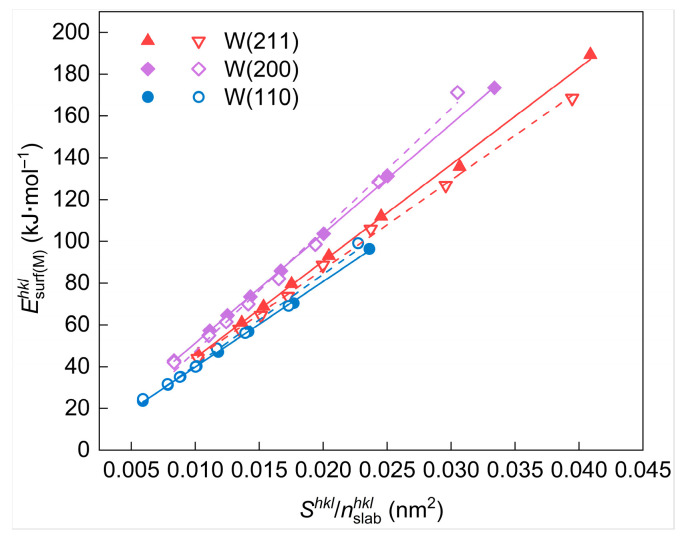
Surface energy $E_{\mathrm{surf}(M)}^{hkl}$ (in unit of kJ/mol) of different W crystal planes as a function of $S^{hkl}/n_{\mathrm{slab}}^{hkl}$. The solid and dashed lines represent the linear fits of $E_{\mathrm{surf}(M)}^{hkl}$ and $S^{hkl}/n_{\mathrm{slab}}^{hkl}$ before and after geometry optimization, respectively.

First of all, the calculation formula of $E_{\mathrm{surf}(M)}^{hkl}$ should be analyzed. It can be revealed through calculation and analysis that $E_{\mathrm{surf}(M)}^{hkl}[\mathrm{kJ}\cdot {\mathrm{mol}}^{-1}]$ is linearly correlated with the ratio of $S^{hkl}$ and $n_{\mathrm{slab}}^{hkl}$ (i.e., $S^{hkl}/n_{\mathrm{slab}}^{hkl}$), as described in Equation (26) and Figure 9. The slope *K* in the equation is a constant value dependent only on the selected crystal plane, indicating that $S^{hkl}/n_{\mathrm{slab}}^{hkl}$ affects the calculated value of $E_{\mathrm{surf}(M)}^{hkl}[\mathrm{kJ}\cdot {\mathrm{mol}}^{-1}]$ at the apparent level.

**Figure 10:**
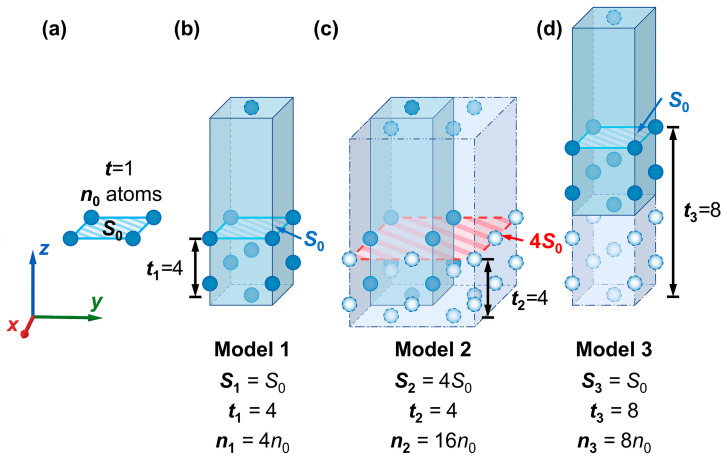
Schematics of surface slab models constructed with different parameters. (**a**) model of the W(110) crystal plane, while the cleavage thickness is the minimum, and the surface area is equal to $S_{0}$, the smallest repeat unit ($n_{0}$—the number of atoms in the smallest repeat unit of a monolayer); (**b**) model of the W(110) crystal plane, with the number of atomic layers $t_{1}=4$ and surface area $S_{1}=S_{0}$, denoted as Model 1; (**c**) Model 1 after four times area expansion in the *XOY* plane, denoted as Model 2; (**d**) Model 1 with an increase in the number of atomic layers to $t_{3}=8$ after increasing the cleavage thickness, denoted as Model 3. Atoms of different colors represent the relationship between the number and distribution of atoms in different models.

It is clear that $S^{hkl}/n_{\mathrm{slab}}^{hkl}$ does not accurately reflect the actual influencing factor for the surface energy measured in kJ/mol. Therefore, the elements $S^{hkl}$ and $n_{\mathrm{slab}}^{hkl}$ of the independent variable $S^{hkl}/n_{\mathrm{slab}}^{hkl}$ are decomposed, analyzed, and combined with Equations (2) and (4) to clarify these influencing factors. Figure 10 shows a brief schematic diagram of the effect of parameter selection on the surface slab model during construction, with the W(110) crystal plane as an example. The conclusion below is also suitable for other crystal planes of W.

After the cleavage operation along the W(110) crystal plane on the W conventional unit cell, the obtained surface can be considered the smallest possible repeat unit in the W(110) plane. The corresponding surface area (i.e., the contact surface area between the vacuum and outermost atomic layers) is considered $S_{0}^{110}$ (simplified here as $S_{0}$). Meanwhile, if the cleavage is performed at the minimum fractional thickness, the resultant model only contains a monolayer of atoms, where the number of the atomic layer t=1, in which the number of atoms contained is denoted as $n_{0}^{110}$ (simplified as $n_{0}$), as illustrated in Figure 10a. A surface slab model constructed with only a monolayer is not accurate enough for practical calculations. Therefore, extending the cell size and increasing the cleavage thickness ($t_{\mathrm{cleave}}$) are expected to improve the accuracy. During the model construction process, as the cleavage thickness increases, the number of atomic layers (*t*) and the total number of atoms ($n_{\mathrm{slab}}$) would also increase. For ease of representation, the values of $S^{110}$, t, and $n_{\mathrm{slab}}^{110}$ for Model 1 to Model 3 in Figure 10 are denoted as $S_{k}$, $t_{k}$, and $n_{k}$, where *k* = 1, 2, 3. As shown in Figure 10b, $t_{1}=4$ and $n_{1}=4n_{0}$ in Model 1. Model 3 can be obtained by increasing the cleavage thickness of Model 1 (shown in Figure 10d). For Model 3, $t_{3}=8$ and $n_{3}=8n_{0}$. The “*Supercell*” command for area expansion is also applied in model construction, which could simultaneously enhance the value of the surface area $S^{hkl}$ and the total number of atoms $n_{\mathrm{slab}}^{hkl}$ in the model. Then, the number of repetitions of the smallest repeating unit in the calculation, *j*, equals the product of the number of repetitions in the *x*- and *y*-directions.

For example, Model 1 is expanded to four times its original area in the *XOY* plane and changed into Model 2 in Figure 10c. At this point, j=4, and $S_{2}=4S_{0}$. Also, $t_{2}=4$, and $n_{2}=4\times 4n_{0}=16n_{0}$. Hence, the relationship between $S^{hkl}$, $n_{\mathrm{slab}}^{hkl}$, and $S_{0}^{hkl}$,$n_{0}^{hkl}$ can be expressed by Equations (27) and (28).

(27) $$S^{hkl}=j\cdot S_{0}^{hkl}$$

(28) $$n_{\mathrm{slab}}^{hkl}=j\cdot n_{0}^{hkl}\cdot t$$

**Figure 11:**
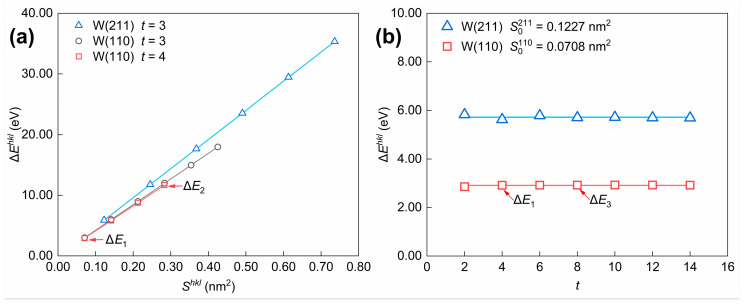
Influence of selected parameters (**a**) $S^{hkl}$ and (**b**) t for model construction on the energy difference ($\Delta E^{hkl}$). The calculation results are derived from the surface slab model for W(110) and W(211) crystal planes. The $\Delta E_{k}$ values of Model 1 to Model 3 in Figure 10 are denoted as $\Delta E_{1}$, $\Delta E_{2}$, and $\Delta E_{3}$.

The term $E_{\mathrm{slab}}^{hkl}-n^{hkl}E_{\mathrm{bulk}}$ (i.e., $E_{\mathrm{slab}}^{hkl}-(n_{\mathrm{slab}}^{hkl}/2)E_{\mathrm{bulk}}$ according to Equation (3)) is present in the numerators of both Equations (2) and (4). The $E_{\mathrm{slab}}^{hkl}-n^{hkl}E_{\mathrm{bulk}}$ values of the three models in Figure 10 (Model 1 to Model 3) are referred to as $\Delta E_{k}^{hkl}$(*k* = 1, 2, 3), representing the energy difference between the atoms in the surface and the bulk. Since the following conclusions hold for the same (*hkl*) crystal plane, $\Delta E_{k}^{hkl}$, $S_{k}^{hkl}$, and $n_{\mathrm{slab}}^{hkl}$ are simplified to $\Delta E_{k}$, $S_{k}$, and $n_{k}$ in Figure 10 and Figure 11, and Table 2. Considering the influence of selected parameters on energy differences in the model construction shown in Figure 11, the analysis of the influence of $S^{hkl}$ and t on $\Delta E^{hkl}$ can be concluded as follows. For the W(110) crystal plane, the relationship between $\Delta E^{hkl}$ and $S^{hkl}$ is depicted in Figure 11a, with the number of layers (t) selected as a constant value. When comparisons are made for surface slab models cleaved along the same crystal plane, the positive relationship between the energy difference ($\Delta E^{hkl}$) and the surface area ($S^{hkl}$) can be shown; in other words, the value of $\Delta E^{hkl}/S^{hkl}$ remains relatively constant. As shown in Figure 11b, the value of $\Delta E^{hkl}$ fluctuates slightly and gradually approaches a constant value as *t* increases when $S^{hkl}$ is certain.

According to the conclusions above, the relationship between the $\Delta E_{k}$ values of the three models in Figure 10b–d can be inferred as shown below. Since the area of Model 1 is equal to that of Model 3, in the meantime, the number of atomic layers in the two models follows a multiple relationship, resulting in $\Delta E_{1}\approx \Delta E_{3}$. Model 2 has the same area as Model 1 but four times the number of atomic layers, so we have $\Delta E_{2}\approx 4\Delta E_{1}$. By substituting the above relationships into Equations (2) and (4), the surface energies in different units of measurement are obtained and listed in Table 2.

As shown in Table 2, $E_{\mathrm{surf}(s),1}^{110}\approx E_{\mathrm{surf}(s),2}^{110}\approx E_{\mathrm{surf}(s),3}^{110}$ indicates that the surface energy value in J/m^2^ could be considered a constant value, consistent with the conclusion in the previous section. In contrast, the values of surface energies in kJ/mol for Models 1–3 are not all equal. According to Figure 10b–d, $t_{1}=t_{2}=\frac{1}{2}t_{3}$, while $E_{\mathrm{surf}(M),1}^{110}\approx E_{\mathrm{surf}(M),2}^{110}\approx 2E_{\mathrm{surf}(M),3}^{110}$ by Equation (4). Therefore, it can be assumed that the value of the surface energy (measured in kJ/mol) is proportional to the inverse of the number of atomic layers in the slab model, meaning that $E_{\mathrm{surf}(M),1}^{110}/\left(1/t_{1}\right)$≈$E_{\mathrm{surf}(M),2}^{110}/\left(1/t_{2}\right)$≈$E_{\mathrm{surf}(M),3}^{110}/\left(1/t_{3}\right)$. Then, the equation for $E_{\mathrm{surf}(M)}^{hkl}$ (i.e., Equation (4)) should be modified as follows (Equation (29)):

(29) $$E_{\mathrm{surf}(M)}^{hkl}=C^{\prime }\cdot \frac{\Delta E^{hkl}}{n_{\mathrm{slab}}^{hkl}}=C^{\prime }\cdot \frac{\Delta E_{0}^{hkl}}{n_{0}^{hkl}t}$$

The variable $n_{0}$ in Equation (29) belongs to the smallest repeat unit in the (*hkl*) surface, as shown in Figure 10a, exhibiting determined values. $\Delta E_{0}^{hkl}$ represents the constant value that $\Delta E^{hkl}$ consistently approaches at different *t* values when the surface area is considered $S_{0}$. Meanwhile, $C^{\prime }$ is a constant. Hence, the $E_{\mathrm{surf}(M)}^{hkl}$ value only depends on the number of atomic layers (*t*). Based on the given information, it is highly likely that the dependence of the $E_{\mathrm{surf}(M)}^{hkl}$ value on the arbitrarily chosen $S^{hkl}$ and $n_{\mathrm{slab}}^{hkl}$ is essentially the dependence of $E_{\mathrm{surf}(M)}^{hkl}$ on *t*.

This conclusion would also be interpreted from the definition of surface energy. The composition and structure of the crystal surface are different from those of the inside of the crystal. It is only beyond several atomic layers along the thickness direction that these properties are essentially similar to those of the internal area [27]. Therefore, for the surface slab model, there is a dividing line along the thickness direction, separating the atoms into “the surface layer atoms” and “the internal atoms”. The change in total energy of the system derived from the formation of surfaces (i.e., the cleavage of crystal planes), which corresponds to the surface energy, is mainly provided by the atoms in the surface layer (i.e., the surface layer atoms), as opposed to the internal atoms. Therefore, it is necessary to identify the delineation described above. Based on the number of atomic layers in the surface layer ($t_{0}$) determined by this delineation, an appropriate and reliable value of $E_{\mathrm{surf}(M)}^{hkl}$ in kJ/mol can be calculated.

To determine the surface layer atoms, the EFS potential function of W metal was modified by extending the nearest neighbor range for further calculations. Then, the total energy per atom before and after cleavage was calculated separately utilizing the modified potential function. The atoms making a notable contribution to the energy change through cleavage can be identified as “the surface layer atoms”. The relevant derivation process is described in detail in Section 2.4. By fitting with the experimental values of physical properties, including pressure (P), bulk modulus ($B_{m}$), shear elastic modulus ($C_{p}$), and elastic constant ($C_{44}$) listed in Table 3 [28], it is feasible to obtain suitable values of the potential parameters. The optimal potential parameters were obtained by fitting the values calculated using Equations (20)–(23), as shown in Table 4. Except for W metal, the same fitting-derivation method was adopted to determine the potential parameter values for Fe metal, thereby validating the universality of the selection method for the surface layer.

Applying the resulting optimal EFS potential parameter values, molecular dynamics calculations were carried out for lattice constant (*a*), cohesive energy ($E_{\mathrm{coh}}$) and vacancy formation energy ($E_{\mathrm{vac}}$) of W and Fe. As presented in Table 5, the calculated values are consistent with the data obtained from experiments [28,29], further confirming the reasonability of the obtained parameters.

After obtaining the optimal potential parameters, calculations were applied to determine the influence of the nearest-neighbor range. Considering the interatomic interactions of the first to k-th nearest neighbor atoms, the energy change per atom before and after cleavage along the (*hkl*) crystal plane can be noted as $\Delta u_{s}^{hkl}(k)$ (*k* = 2,3,4). The variation in $\Delta u_{s}^{hkl}(k)$ values ($\Delta_{k,k+1}\left(u_{s}^{hkl}\right)$) obtained by considering different nearest-neighbor ranges was calculated by Equations (24) and (25) and shown in Table 6.

According to the results, $\left|\Delta_{23}\left(u_{s}^{hkl}\right)\right|$ is significantly larger than $\left|\Delta_{34}\left(u_{s}^{hkl}\right)\right|$, suggesting that the energy change caused by surface cleavage mainly originates from the interatomic interactions of the first to third nearest neighbor atoms. At the same time, those of the fourth nearest neighbor atoms are relatively negligible. In consequence, the main contribution to the surface energy calculated based on the energy change by cleaving is provided by atoms within the third nearest-neighbor distance. The relationship between interatomic interactions and the nearest-neighbor range, as calculated using the potential function, remains valid for other metals in addition to tungsten, indicating that the surface layer selection method employed in this work possesses universality. It should be mentioned that the distance between the reference atom and its third nearest neighbor atom ($r_{3}=\sqrt{2}a$ = 0.4476 nm) is twice the interplanar spacing of the W(110) crystal plane ($d_{110}$ = 0.2238 nm). Moreover, the line between the reference atom and its third nearest neighbor atom is parallel to the <110> direction. As a result, the selection of the number of surface atomic layers ($t_{0}$) in the surface slab models for {110} crystal plane family surfaces and non-{110} crystal plane family surfaces is discussed separately. The detailed process is described as follows.

**Figure 12:**
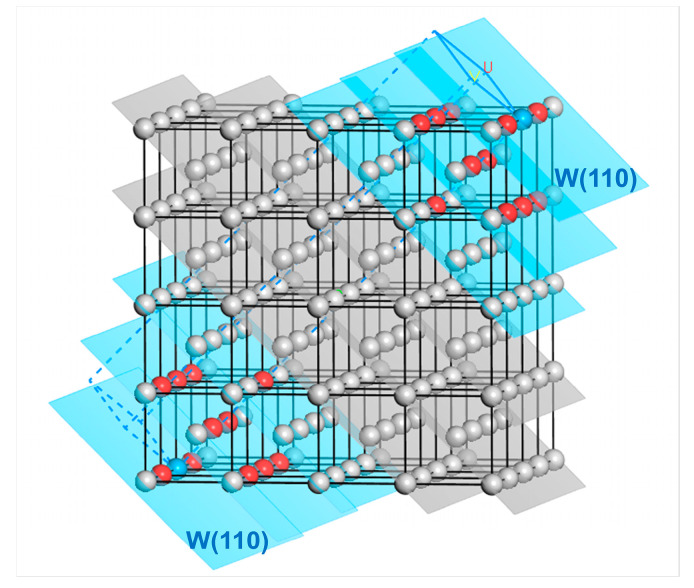
Selection of the number of atomic layers in the surface layer on both sides for the surface slab model of the W(110) crystal plane. The red-colored atoms are the first to third nearest neighbor atoms of the outermost reference atoms (blue), while the gray atoms are those whose distance from the reference atom exceeds the third nearest-neighbor distance. The surface layer atoms are located in the blue-colored W(110) crystal planes. The letters U and V represent the surface vectors during cleavage.

Taking the W(110) surface as an example, the cleavage process is shown in Figure 12. The outermost atoms at both ends are regarded as the reference atoms (colored in blue). In contrast, all the atoms within their third nearest neighbor range are selected as the surface layer atoms (colored in red). These atoms are located in the three W(110) layers (the blue-colored planes in Figure 12), including the outermost one. At this point, the corresponding number of atomic layers in the surface layer is $t_{0}=6$ for the two W(110) surfaces formed by cleavage.

The method for selecting $t_{0}$ values used to calculate the surface energy of any {*hkl*} crystal plane in kJ/mol can be summarized as a calculation formula, as described below. The line between the reference atom and its third nearest neighbor atom is always perpendicular to the crystal planes in the {110} crystallographic family, but not always perpendicular to the (*hkl*) crystal plane for which the surface energy is to be calculated. At this point, a correction factor $\cos\theta_{0}$ should be introduced, representing the maximum value of the cosine of the angle (cosθ) between the normal direction of the $\left(h_{2}k_{2}l_{2}\right)$ crystal plane for which the surface energy is to be calculated and the <110> crystal direction. In this work, the $\cos\theta_{0}$ value is calculated using the formula for the angle between the crystal planes in the cubic crystal system:

(30) $$\cos\theta_{0}={\left(\frac{h_{1}h_{2}+k_{1}k_{2}+l_{1}l_{2}}{\sqrt{h_{1}^{2}+k_{1}^{2}+l_{1}^{2}}\sqrt{h_{2}^{2}+k_{2}^{2}+l_{2}^{2}}}\right)}_{\max}(<h_{1}k_{1}l_{1}>=<110>)$$

The third nearest-neighbor distance $r_{3}$ is projected onto the normal direction of $\left(h_{2}k_{2}l_{2}\right)$ crystal plane. Then the resulting projected distance $r_{3}\cos\theta_{0}$ is divided by the interplanar spacing $d_{h_{2}k_{2}l_{2}}$ of $\left(h_{2}k_{2}l_{2}\right)$ crystal plane to obtain the number of atomic layers $T^{\prime }$ corresponding to the constructed surface slab model. Therefore, the calculation formula is expressed as

(31) $$T^{\prime }=\frac{r_{3}\cos\theta_{0}}{d_{h_{2}k_{2}l_{2}}}$$

**Figure 13:**
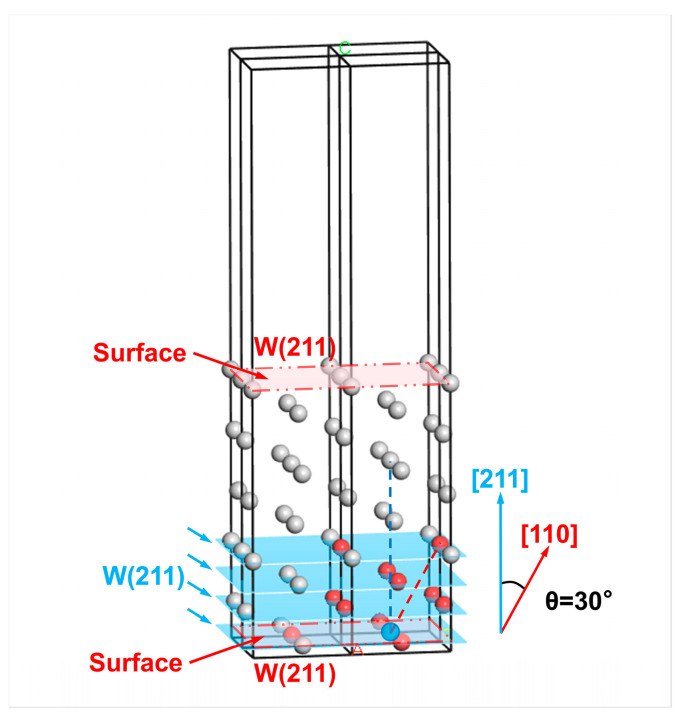
Selection of the surface layer atoms on one side of the W(211) surface in the slab model based on the third nearest-neighbor distance range. The red-colored atoms are the first to third nearest neighbor atoms of the outermost reference atom (blue), while the gray atoms are those whose distance from the reference atom exceeds the third nearest-neighbor distance. The atoms in the blue-colored W(211) crystal planes are denoted as the surface layer atoms. The different letters represent labeled axes, defining the crystal directions corresponding to the atomic arrangements in the model.

For example, the selection of surface layer atoms of a W(211) surface is shown in Figure 13. The crystal plane angle satisfies $\cos\theta_{0}=0.8660$, i.e., $\theta_{0}=30^{\circ }$. Then, the number of atomic layers in the surface layer should equal the projection $r_{3}\cos\theta_{0}$ of the third nearest-neighbor distance $r_{3}$ in the direction normal to the W(211) crystal plane (i.e., $0.8660r_{3}$) divided by the interplanar spacing $d_{211}$ of the W(211) crystal plane. Since the calculations of surface energies are based on the surfaces at both ends of the slab model, the final selected number of atomic layers in the surface layers meets $t_{0}=2\left(\left\lceil T^{\prime }\right\rceil +1\right)=2\left\lceil T^{\prime }\right\rceil +2$, where $\left\lceil T^{\prime }\right\rceil$ represents the smallest integer greater than or equal to $T^{\prime }$ [30]. The relationship between $t_{0}$ and the crystal plane index can be summarized as follows:

(32) $$t_{0}=2\left\lceil \frac{r_{3}}{d_{h_{2}k_{2}l_{2}}}\cdot {\left(\frac{h_{1}h_{2}+k_{1}k_{2}+l_{1}l_{2}}{\sqrt{h_{1}^{2}+k_{1}^{2}+l_{1}^{2}}\sqrt{h_{2}^{2}+k_{2}^{2}+l_{2}^{2}}}\right)}_{\max}\right\rceil +2$$

It should be noted that there is an overlap in the range of the third nearest-neighbor distance for several high-index crystal planes. In these circumstances, the number of atomic layers in the surface layer should be selected as $t_{0}=2\left\lceil T^{\prime }\right\rceil$. According to the selection scheme for the third-nearest-neighbor range, the number of atomic layers in the surface layer $t_{0}$ and the corresponding total number of atoms $n_{\mathrm{slab}}^{hkl}$ can be determined. Upon substituting the $n_{\mathrm{slab}}^{hkl}$ values into Equation (4), the calculated $E_{\mathrm{surf}(M)}^{hkl}$ values (in kJ/mol) can be considered the required values, as shown in Table 7.

### 3.3. Mechanisms for the Impact of Surface Energy on Surface Activity

**Figure 14:**
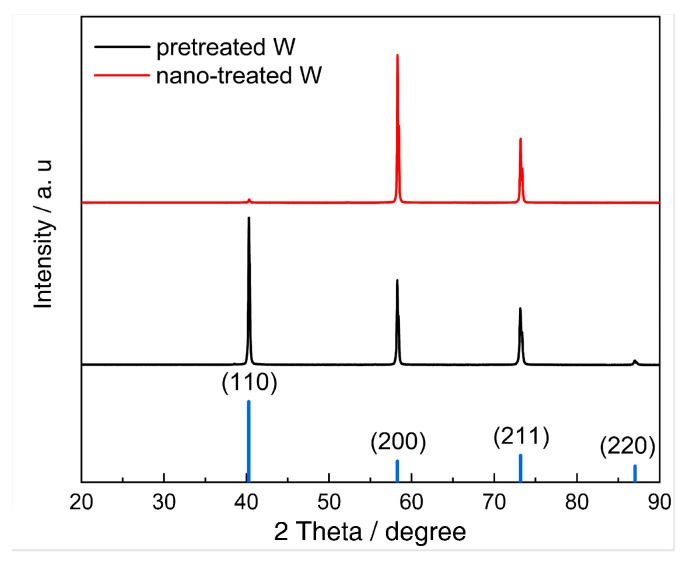
The XRD patterns of the W samples before and after nano-treatment.

As demonstrated by the experimental results in Section 3.1, the effective enhancement of the surface activity is achieved using the nanosized method developed in this work. Moreover, the W samples annealed at 600 °C exhibited optimal activity. The thermodynamically characterized surface energies of each crystal plane, expressed in kJ/mol, were calculated in Section 3.2. Based on the results above, the reasons for the enhancement of surface activity can be further investigated through computational analysis. The total surface energies of W samples (measured in kJ/mol) can be calculated before and after the nano-treatment, using Equation (33) [7].

(33) $$E_{\mathrm{surf}(M)}=\sum_{hkl}f_{hkl}E_{\mathrm{surf}(M)}^{hkl}$$

where $E_{\mathrm{surf}(M)}^{hkl}$ is the surface energy of the (*hkl*) crystal plane in units of kJ/mol, while $f_{hkl}$ represents the ratio of crystal planes measured by XRD tests, with the results shown in Figure 14. The ratio and calculated surface energy of each crystal plane, as well as the total surface energies of pretreated W and nano-treated W, are listed in Table 8.

The nanoporous structure can be approximated as a structure made up of interconnected ligaments and pores at the nanometer scale [31]. These metallic ligaments can be obtained through etching, the inverse process of crystal surface growth [32], which results in the exposure of more high-surface-energy crystal planes, in contrast to the formation of planes with low surface energies during the crystal growth process. The surfaces exposed after etching are not entirely parallel to the macroscopic surface and may have a significant angle difference. However, due to the numerous metal ligaments and their random distribution in all directions of equal probability, the ligament orientations in which the crystal planes constructing the surface of the ligament (denoted as $\left\{h^{\prime }k^{\prime }l^{\prime }\right\}$) conform to the Bragg equation always exist, which would be approximated as X-ray powder diffraction [33]. Moreover, the ligament orientations in which these $\left\{h^{\prime }k^{\prime }l^{\prime }\right\}$-planes are involved in X-ray diffraction could reflect the composition and content of planes in other ligament orientations in which the $\left\{h^{\prime }k^{\prime }l^{\prime }\right\}$-planes are not involved. Therefore, the content of crystal planes involved in the XRD tests can be approximated to reflect that of different crystal planes throughout the nanostructure. In other words, Equation (33) is applicable for calculations of NPW synthesized in this work.

As shown in Table 8, the average surface energy of NPW (annealed at 600 °C, with optimal morphology) was enhanced by 23.15% compared to that without nano-treatment. Combined with the preceding electrochemical test results, it can be inferred that the novel nanosized method applied in this work effectively improves the total surface energy and, consequently, the surface activity. Diffraction extinction crystal planes would not affect the surface energy calculation results based on XRD diffraction. This is because diffraction extinction crystal planes (extinction under conditions where *h + k + l* = odd) share the same interplanar spacing and surface energy with non-extinction crystal planes whose Miller indices are even multiples of the extinction planes. The proportion of diffraction extinction crystal planes can be represented by the proportion of non-extinction crystal planes with identical interplanar spacing and surface energy. Consequently, the law of influence of the non-extinction crystal planes on surface energy is similar to that of the corresponding diffraction extinction crystal planes. In summary, this method enables qualitative analysis of the influence of surface energy on activity. At the same time, the results also confirm the validity of the surface energy calculation method we proposed for correlated energy calculation and analysis applications in units of kJ/mol.

## 4. Conclusions

(1) A nanoporous layer with uniformly distributed pores was synthesized by etching and annealing via the novel nanosized method. The enhancement of surface activity by this nano-treatment was confirmed by electrochemical tests.
(2) A novel computational method for surface energy calculations was proposed based on first-principles computations, converting the unit of measurement from J/m^2^ to kJ/mol, which allows the resulting surface energies to exhibit thermodynamic characteristics. It was confirmed by the analysis that the selected number of atomic layers (*t*) significantly influences the value of surface energies measured in kJ/mol. The resulting surface energy value in new units is relatively appropriate when *t* equals *t*_0_, the number of atomic layers in the surface layer. To obtain the appropriate $t_{0}$ values, the energy change of a single atom caused by cleavage, considering different nearest-neighbor ranges, was calculated through the improved EFS potential function, subsequently confirming that the effect of the fourth nearest neighbor interatomic interactions on the energy change was negligible. This conclusion also holds for other metals except W. Based on the findings above, the range of the surface layer can be determined by selecting the number of atomic layers corresponding to the first to third nearest neighbors. Consequently, a set of referable $t_{0}$ values and corresponding first-principles surface energy values (in units of kJ/mol) was given, providing a solution for the indeterminable number of selected atomic layers and surface energy values measured in kJ/mol.
(3) These calculated surface energies of different crystal planes measured in kJ/mol, combined with the content of crystal planes determined by XRD tests, were utilized to calculate and compare the total surface energy before and after nano-treatment. The calculation results confirm that nano-treatment can enhance surface energy, which can be considered an activity origin. Meanwhile, the feasibility of the surface energy calculation method is validated by the obtained results.

## Figures and Tables

**Table 1:** Surface energy values of different W crystal planes before and after geometry optimization, expressed in J/m^2^.

State	W(110)	W(200)	W(211)	W(420)	W(222)	W(442)
Before optimization	3.305	3.726	4.269	4.003	3.961	3.936
After optimization	3.472	3.540	4.123	3.584	3.868	3.741

**Table 2:** Surface area ($S_{k}$), number of atomic layers ($t_{k}$), total number of atoms ($n_{k}$; *k* = 1, 2, 3), and the calculated surface energy values ($E_{\mathrm{surf}(s),k}^{110}$ in J/m^2^ and $E_{\mathrm{surf}(M),k}^{110}$ in kJ/mol) for surface slab models (Model 1~Model 3 in Figure 10b–d).

Model	$S_{k}$	$t_{k}$	$n_{k}$	$E_{\mathrm{surf}(S),k}^{110}$/(J/m^2^)	$E_{\mathrm{surf}(M),k}^{110}$/(kJ/mol)
1	$S_{0}$	4	$4n_{0}$	$\Delta E_{1}/2S_{0}$	$C^{\prime }\Delta E_{1}/4n_{0}$
2	$4S_{0}$	4	$16n_{0}$	$\approx \Delta E_{1}/2S_{0}$	$\approx C^{\prime }\Delta E_{1}/4n_{0}$
3	$S_{0}$	8	$8n_{0}$	$\approx \Delta E_{1}/2S_{0}$	$\approx C^{\prime }\Delta E_{1}/8n_{0}$

$C^{\prime }=CN_{A}$, $C^{\prime }$—unit conversion factor to kJ/mol; $N_{A}$—Avogadro constant; C(=1.60 × 10^−19^ J/eV)—unit conversion factor from electron volt (eV) to joule (J).

**Table 3:** Experimental values of physical properties of bcc metals utilized in the fitting operation [28].

Metal	P (10^5^ MPa)	$B_{m}$ (10^5^ MPa)	$C_{44}$ (10^5^ MPa)	$C_{p}$ (10^5^ MPa)
W	0	3.1420	1.6310	1.6381
Fe	0	1.6867	1.1600	0.4300

P—pressure; $B_{m}$—bulk modulus; $C_{p}$—shear elastic modulus; $C_{44}$—elastic constant.

**Table 4:** The optimal potential parameters obtained by fitting with physical properties.

Metal	*A* (10 eV·nm^−1^)	*d* (nm)	*c* (nm)	*c*_0_ (10^2^ eV·nm^−2^)	*c*_1_ (10^3^ eV·nm^−3^)	*c*_2_ (10^4^ eV·nm^−4^)	*c*_3_ (10^5^ eV·nm^−5^)	*c*_4_ (10^6^ eV·nm^−6^)	*B* (10^2^ nm^−2^)
W	0.8670	0.53826	0.52987	6.2896	−6.7674	2.8083	−0.5326	0.0386	0.2681
Fe	0.5420	0.49634	0.47948	3.8753	−4.2391	1.7077	−0.3009	0.0198	0.0420

*A*, *d*, *c*, *c*_0_, *c*_1_, *c*_2_, *c*_3_, *c*_4_, *B*—potential parameters.

**Table 5:** Comparison of the computed and experimental values [28,29] of lattice constant, cohesive energy, and vacancy formation energy of W and Fe. The computed values are obtained based on the developed EFS potential with the potential parameters in Table 4.

Metal	*a* (nm)	*E*_coh_ (eV)	*E*_vac_ (eV)	Ref.
W	0.3165	8.899	3.952	This work
	0.3165	8.900	3.950	Experimental [28,29]
Fe	0.2869	4.297	1.807	This work
	0.2870	4.280	1.790	Experimental [28,29]

Note: *a*—lattice constant, *E*_coh_—cohesive energy, *E*_vac_—vacancy formation energy.

**Table 6:** The computed values of the energy change for each W atom and Fe atom before and after cleavage along the (*hkl*) crystal plane, considering different nearest-neighbor ranges ($\Delta u_{s}^{hkl}(k)$). Calculations are applied using the potential parameters in Table 4. $\Delta_{k,k+1}\left(u_{s}^{hkl}\right)$ represents the variation in $\Delta u_{s}^{hkl}(k)$ and $\Delta u_{s}^{hkl}(k+1)$. For $\Delta u_{s}^{hkl}(k)$, the unit of measurement is standardized to kJ·mol^−1^. The nearest neighbor number *k* = 2, 3, 4.

Metal	(hkl)	$\Delta u_{s}^{hkl}\left(2\right)$ (kJ·mol^−1^)	$\Delta_{23}\left(u_{s}^{hkl}\right)$	$\Delta u_{s}^{hkl}\left(3\right)$ (kJ·mol^−1^)	$\Delta_{34}(u_{s}^{hkl})$	$\Delta u_{s}^{hkl}\left(4\right)$ (kJ·mol^−1^)
W	W(200)	171.473	−10.086%	190.707	−0.010%	190.727
	W(110)	111.892	−12.759%	128.257	−0.023%	128.286
	W(222)	353.247	−6.230%	376.715	−0.001%	376.720
Fe	Fe(200)	82.450	−4.450%	86.290	−1.962%	88.017
	Fe(110)	47.604	−16.314%	56.884	−2.110%	58.111
	Fe(222)	116.996	−31.147%	169.922	−1.622%	172.723

**Table 7:** Surface energies of different W crystal planes (measured in kJ/mol). The corresponding energies after geometry optimization are presented in parentheses.

Crystal Plane	(110)	(200)	(211)	(222)	(420)	(442)
$t_{0}$	6	6	8	10	12	16
$n_{\mathrm{slab}}$	6	6	8	10	12	16
$E_{\mathrm{surf}(M)}^{hkl}$ (kJ/mol)	46.988 (48.843)	85.849 (82.085)	68.824 (64.523)	84.982 (76.446)	89.043 (84.623)	89.036 (83.243)

$t_{0}$—the number of atomic layers in the surface layer; $E_{\mathrm{surf}(M)}^{hkl}$—surface energies of different W crystal planes measured in kJ/mol.

**Table 8:** The ratio of crystal planes and surface energies of different crystal planes before and after nano-treatment (in kJ/mol).

Crystal Plane	(110)	(200)	(211)	(220)	$E_{\mathrm{surf}(M)}$(kJ/mol)
$f_{hkl}$/%(pretreated W)	48.48%	29.52%	20.46%	1.54%	61.86
$f_{hkl}$/%(nano-treated W)	1.35%	67.60%	31.05%	0	76.18
$E_{\mathrm{surf}(M)}^{hkl}$(kJ/mol)	48.84	82.09	64.52	48.84	-

$f_{hkl}$—ratio of crystal planes, $E_{\mathrm{surf}(M)}$—total surface energy measured in kJ/mol.

## Data Availability

The original contributions presented in this study are included in the article. Further inquiries can be directed to the corresponding author.
